# Taxonomic and enzymatic basis of the cellulolytic microbial consortium KKU-MC1 and its application in enhancing biomethane production

**DOI:** 10.1038/s41598-023-29895-0

**Published:** 2023-02-20

**Authors:** Nantharat Wongfaed, Sompong O-Thong, Sureewan Sittijunda, Alissara Reungsang

**Affiliations:** 1 Department of Biotechnology, Faculty of Technology, Khon Kaen University, Khon Kaen, 40002 Thailand; 2 International College, Thaksin University, Songkhla, 90000 Thailand; 3 Faculty of Environment and Resource Studies, Mahidol University, Nakhon Pathom, 73170 Thailand; 4 Research Group for Development of Microbial Hydrogen Production Process from Biomass, Khon Kaen University, Khon Kaen, 40002 Thailand; 5 Academy of Science, Royal Society of Thailand, Bangkok, 10300 Thailand

**Keywords:** Biochemistry, Biotechnology, Microbiology, Molecular biology

## Abstract

Lignocellulosic biomass is a promising substrate for biogas production. However, its recalcitrant structure limits conversion efficiency. This study aims to design a microbial consortium (MC) capable of producing the cellulolytic enzyme and exploring the taxonomic and genetic aspects of lignocellulose degradation. A diverse range of lignocellulolytic bacteria and degrading enzymes from various habitats were enriched for a known KKU-MC1. The KKU-MC1 was found to be abundant in *Bacteroidetes* (51%), *Proteobacteria* (29%), *Firmicutes* (10%), and other phyla (8% unknown, 0.4% unclassified, 0.6% archaea, and the remaining 1% other bacteria with low predominance). Carbohydrate-active enzyme (CAZyme) annotation revealed that the genera *Bacteroides,* *Ruminiclostridium,* *Enterococcus*, and *Parabacteroides* encoded a diverse set of cellulose and hemicellulose degradation enzymes. Furthermore, the gene families associated with lignin deconstruction were more abundant in the *Pseudomonas* genera. Subsequently, the effects of MC on methane production from various biomasses were studied in two ways: bioaugmentation and pre-hydrolysis. Methane yield (MY) of pre-hydrolysis cassava bagasse (CB), *Napier* grass (NG), and sugarcane bagasse (SB) with KKU-MC1 for 5 days improved by 38–56% compared to non-prehydrolysis substrates, while MY of prehydrolysed filter cake (FC) for 15 days improved by 56% compared to raw FC. The MY of CB, NG, and SB (at 4% initial volatile solid concentration (IVC)) with KKU-MC1 augmentation improved by 29–42% compared to the non-augmentation treatment. FC (1% IVC) had 17% higher MY than the non-augmentation treatment. These findings demonstrated that KKU-MC1 released the cellulolytic enzyme capable of decomposing various lignocellulosic biomasses, resulting in increased biogas production.

## Introduction

Biogas production from organic waste materials through anaerobic digestion (AD) has attracted worldwide interest in recent years. This technology could meet the increasing energy demand and tackle the environmental pollution problem^1^. The feedstock used for biogas fermentation is abundant; lignocellulosic biomass, such as sugarcane bagasse (SB), and filter cake (FC) from sugar industries*,* also *Napie*r grass (NG), cassava bagasse (CB), and some kinds of industrial waste, are the most common and easily accessible. However, utilizing this lignocellulosic biomass for bioconversion is challenging because of the highly recalcitrant nature of the plant cell wall, which comprises cellulose microfibers bound to hemicellulose networks and protected by lignin. Furthermore, the low hydrolysis rate of lignocellulosic biomass causes the degradation process to slow, reducing methane production efficiency. In order to increase biogas production from lignocellulosic biomass, a pretreatment method is required before further processing^2^. The pretreatment methods used can be classified as physical, chemical, and biological^3^. Physical and chemical pretreatments can disrupt the lignocellulose structure in a very short time, thus enhancing its biodegradability. However, these methods increase the process cost and generate toxic compounds or inhibitors in the environment^4^. In addition, acid or alkali treatments are required for neutralization after pretreatment, complicating the process.

Biological pretreatment, which uses enzymes or microorganisms to prepare lignocellulose biomass for biogas production, can be time-consuming compared to physical and chemical pretreatments. However, these technologies are promising because they are environment-friendly and cost-effective^4^. However, various factors must be carefully controlled to maintain stable or persistent enzymatic activity, such as the type of substrate, pretreatment time, pH, and temperature. According to Parawira, using free enzymes may be less efficient and effective than cultivating microorganisms that produce stable and persistent lignocellulose-degrading compounds^5^. On the other hand, using a mixture of several isolated microorganisms is more effective than using single strains because of the complex nature of lignocellulose^6^. The microbial consortium (MC) can be isolated from various ecological niches, including forest compost soil^7^, compost habitats^8^, SB compost^9^, and composting under AD^10^. Several studies have successfully used MC to improve the biodegradation of lignocellulosic biomass and increase biogas production. For example, Wongwilaiwalin et al. found that NG treated with MC (created from seed cultures harvested from degrading bagasse compost) for 7 days increased the methane yield (MY) by 37% compared to untreated NG^11^. In addition, MC enriched from compost, plant litter, and animal and agricultural wastes enhanced the methane production from NG^12^.

The taxonomic study of genes involved in lignocellulose degeneration is crucial because it allows us to link specific functions to various microorganisms and identify potential synergistic characteristics^13^. Thus, metagenomic approaches have been beneficial in studying the biodegradative potential of microbial consortia and the genes encoding enzymes that hydrolyze plant polymers. Functional genes involved in carbohydrate degradation have been investigated in numerous studies. For example, Zhu et al. explored the taxonomic and enzymatic basis of the lignocellulose-degrading ability of the corn stover‑adapted microbial consortium EMSD5^8^. Colombo et al. discovered genes encoding carbohydrate-depleting enzymes from an MC derived from a thermophilic composting phase of SB and cow manure^14^. Oh et al. explored the selective lignocellulose-degrading enzyme capability of certain bacteria that acted on wood detritus^15^. These studies help in understanding the function of microbes. Moreover, taxonomic studies of the MC that respond to different complex substrates can provide information on their specific enzyme systems^16^.

This research evaluated two strategies for utilizing the MC to improve methane production from lignocellulosic biomass. Bioaugmentation utilizes microbial isolates, co-cultures, or complex communities, which is an environment-friendly and cost-effective strategy for increasing biogas production^17^. Bioaugmentation in anaerobic digesters can be accomplished by adding pure species or MC. MC is preferable to pure culture because MC contains complex enzymes required for degrading lignocellulosic biomass and can effectively improve the degradation rate^17^. Bioaugmentation can increase biogas production, and numerous studies in the literature confirm this. For instance, Yu et al. used MC1 to accelerate the acidification of corn stalks and cow dung and improved the MY by 22.1% compared to that obtained after natural acidification^18^. Weiss et al. augmented maple leaves and wheat straw using MC (enriched from hydrolytic microorganisms from biogas sludge), producing a significantly higher MY (42% higher than the control)^19^. Moreover, many studies have reported that augmentation increases methane production from cellulosic waste^13,20,21^. The second method is to use MC to pre-hydrolyze lignocellulosic material. Since the MC has a relationship between various microorganisms and their lignocellulolytic enzyme systems, it is helpful to design enzyme cocktails based on enzyme interaction. The MC depolymerizes the macromolecules to oligo- and monomers during the pre-hydrolysis stage via extracellular enzyme secretion, resulting in the release of dissolved compounds that are easily accessible to methanogenic archaea during the AD process^22^. The pre-hydrolysis step has been studied in AD to see if it can increase the hydrolysis of complex substrates^21^. Suksong et al. discovered that a consortium rich in *Lachnospiraceae* and *Clostridiaceae* improved hydrolysis and acidogenesis of oil palm empty fruit bunches (EFB), resulting in improved EFB degradation efficiency when compared to untreated EFB^21^, resulting in a two to tenfold increase in MY of EFB pre-hydrolysis. Furthermore, pre-hydrolysis of rice straw for 14 days with an anaerobic lignocellulolytic MC increased MY by 53% compared to untreated rice straw^23^. Thus, in this study, bioaugmentation and pre-hydrolysis strategies were used to hydrolyze lignocellulosic materials and improve AD efficiency using MC. The aim of this research was to design the MC capable of producing the cellulolytic enzyme. MCs' structure, functions, and metabolic pathways were investigated using metagenomic analysis. Furthermore, the MC was used to determine the best strategy (bioaugmentation and pre-hydrolysis) for increasing biogas production from various biomasses with varying initial volatile solid concentrations (IVC).

## Results and discussion

### MC screening and selection

**Figure 1:**
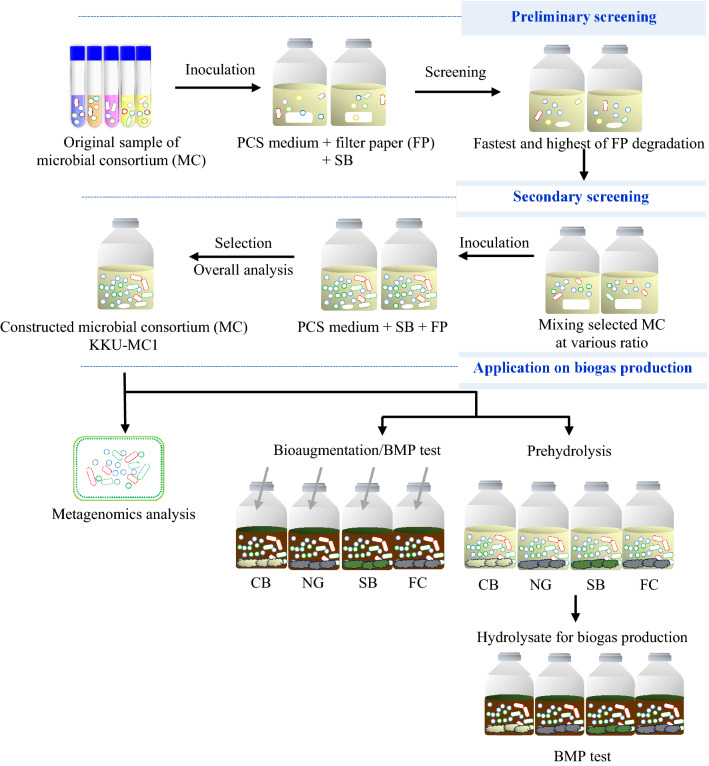
Flow chart of the experiments. *CB* Cassava bagasse, *NG* Napier grass, *SB* sugarcane bagasse, *FC* filter cake, *BMP* biochemical methane potential test, *PCS* peptone cellulose solution.

MC screening and selection were performed as shown in Fig. 1. As a primary screening, the filter paper degradation rate was used to assess the lignocellulose utilization efficiency of the MC. The MC sources that show a rapid filter paper degradation rate can potentially be used for pretreating lignocellulose^24^. Cellulose is the major component, accounting for approximately 37.66 ± 5.5% of the dry weight of SB (Table 1). Therefore, screening cellulose-degrading microorganisms is another crucial step in the rapid degradation of lignocellulosic material. In the preliminary screening results, microorganisms originating from the rice straw compost (RSC), the soil around the goat, sheep stalls (SGS), and termite intestines (TI) had the highest filter paper degradation rates (0.9–1.0 cm/day). This result was consistent with SB's high volatile solid (VS) degradation efficiency (51–52%) (Table 2). The MC from these sources had almost completely degraded filter paper in less than five days with high enzyme activity. Results are consistent with a study by Zhong et al., who found that the screened bacteria showed nearly complete filter paper degradation in 4 days^24^. The production of four enzymes that degrade lignocellulosic biomass was evaluated: cellulase (filter paperase; FPase), endoglucanase (carboxy methyl cellulase; CMCase), exoglucanase (avicelase), and hemicellulases (xylanase). High FPase activity was observed in all microbial sources in the 0.47–0.52 international unit (IU)/mL range. High CMCase, avicelase, and xylanase activities were observed in the RSC, SGS, and TI sources. Different enzyme activity values were associated with the normal flora in each natural source. The remaining microbial sources showed completely degraded filter paper during days 7–8 of digestion and had relatively low CMCase and xylanase activity, which resulted in a low VS degradation efficiency of the SB (43–46%). In the initial screening, three microbial groups that originated from the RSC, SGS, and TI presented high filter paper degradation rates, enzyme activity, and VS removal efficiency of the SB. Hence, the degradability of lignocellulose was selected based on the initial screening test. For the second screening to select the microbial mixtures from different sources, we focus on the symbiotic connections (commensalism and mutualism) in microbial consortia, which drive members to work together and perform various yet complementary functions. The parameters for the secondary selection of microbial mixtures were determined after subculturing 10 times to achieve a stable MC^25^. Generally, the application of MC in biomass pretreatment was focused on cellulose and hemicellulose degradation or lignin removal. Here, we investigate how the enzymatic activity and VS removal efficiency varied at the proportions of RSC, SGS, and TI (Table 3). The statistical analysis at a 95% confidence level (p > 0.05) is shown in Supplementary Table S1. The quadratic and cubic model was chosen as the most effective one for explaining the experimental data (Table S1). The quadratic and cubic model obtained from the mixture design with D-optimal and the analysis of variance (ANOVA) for this model is presented in Supplementary Table S2. To validate the model, an increase in the proportions of SGS resulted in increased FPase activity, while TI does not affect the FPase activity (supplementary Fig.S1a). The avicelase and CMCase may be changes in activities when adjusted the proportion of SGS, which amount should be in the range of 2.0–2.5 mL (supplementary Fig. S1b,c). Additionally, xylanase activity and VS removal efficiency has been strongly affected by the amount of TI, with these values rising as the fraction of SGS increases (supplementary Fig. S1d, S1e). In the meantime, the combination of RSG and SGS is unrelated to improving xylanase activity and VS removal efficiency. From the validation of the model, the combination of RSG, SGS, and TI at optimal proportions 1:1:1 was conducted. The calculation of the combination effect from the response of the mixture design is shown in Table 3. In the case of FPase, the combination effect of Runs 1–16 was in the ranges of 0.22 to 2.03. The combination effect greater than 1 indicates that using a combination of RSC, SGS, and TI at a 1:1:1 ratio (Run 11) is a good way to increase FPase activity. In addition, Run 5 showed a relatively high combination effect (1.98). However, the antagonistic effect of 0.22 on FPase was observed in the proportion of RSC and TI at a 1:1 ratio (Run 6). The synergistic effect on CMCase and avicelase activity was observed in all Runs, especially in Runs 4, 10, and 11. However, Runs 6, 8 (CMCase activity), and 8 (avicelase activity) had a combined effect of 0.9–1.0, approximately the same proportion with neither antagonistic nor synergistic effect. Antagonistic effect on xylanase activity, a combination effect of 0.10–0.12 was obtained from the combination in Run 5 and 7. Meanwhile, a synergistic effect on xylanase activity, a combination effect of 3.13, was found at the optimal proportions of microorganism source, i.e., at a 1:1:1 ratio of combination as RSC, SGS, and TI (Run 11). A synergistic effect on VS removal efficiency was also found in Run 11, with a combined effect of 2.43. The results showed that the combined MC of Run 6 antagonizes all enzymes involved in lignocellulose degradation. In contrast, the combination of MC from Run 11 showed a synergistic effect on enzymes involved in lignocellulose degradation and the efficiency of VS removal. Lin et al. stated that sharing and exchanging public goods such as carbohydrates and aromatic monomers is the essence of cellulolytic microbial synergistic interactions^25^. In our case, the MC1 showed mutualism (+/+), one of the synergistic interactions caused by sharing public goods^26,27^.

**Table 1:** Characteristics of the substrates used in this experiment.

Parameter	Unit	CB	NG	SB	FC
pH	–	4.45 ± 0.01	4.97 ± 0.02	4.64 ± 0.01	7.06 ± 0.01
Moisture	%	10.90 ± 0.17	6.56 ± 1.56	4.83 ± 0.03	2.55 ± 0.36
TS	%	89.10 ± 0.17	93.44 ± 1.56	95.17 ± 0.03	97.45 ± 0.36
VS	%TS	87.37 ± 0.18	83.43 ± 0.00	84.01 ± 0.09	36.07 ± 0.05
Ash	%TS	1.12 ± 0.00	1.07 ± 0.02	1.05 ± 0.00	1.03 ± 0.00
sCOD	% TS	1.25 ± 0.270	0.90 ± 0.250	0.74 ± 0.160	0.64 ± 0.230
Cellulose	% TS	21.88 ± 4.5	35.66 ± 3.5	37.66 ± 5.5	11.52 ± 1.2
Hemicellulose	% TS	14.94 ± 5.6	22.12 ± 2.5	33.72 ± 1.2	7.45 ± 2.5
Lignin	% TS	12.61 ± 5.6	20.05 ± 5.3	20.45 ± 2.2	6.28 ± 1.4

**Table 2:** Degradation efficiency and enzymatic activity of various microbial sources in the primary selection^a^.

Source	Filter paper degradation rate (cm/day)	VS removal efficiency (%)	Enzyme activity (IU/mL)			
			FPase	CMCase	Avicelase	Xylanase
RSC	0.9 ± 0.30	51.95 ± 0.30	0.51 ± 0.25	0.02 ± 0.15	0.04 ± 0.18	0.66 ± 0.20
CM	0.6 ± 0.20	44.25 ± 0.15	0.48 ± 0.22	0.01 ± 0.25	0.02 ± 0.50	0.21 ± 0.21
GD	0.5 ± 0.15	45.85 ± 0.25	0.49 ± 0.15	0.01 ± 0.10	0.02 ± 0.20	0.16 ± 0.13
SGS	0.9 ± 0.15	50.81 ± 0.20	0.52 ± 0.10	0.02 ± 0.26	0.04 ± 0.10	0.41 ± 0.22
RF	0.5 ± 0.20	43.24 ± 0.30	0.47 ± 0.22	0.01 ± 0.25	0.04 ± 0.41	0.21 ± 0.15
TI	1.0 ± 0.10	52.46 ± 0.30	0.49 ± 0.20	0.04 ± 0.20	0.03 ± 0.55	0.52 ± 0.18

^a^All the data in each microbial source was measured when the filter paper complete degradation.

**Table 3:** Mixture experimental design defining proportions of rice straw compost (RSC), soil around goat and sheep stalls (SGS), and termite intestines (TI) and respective values of FPase, CMCase, avicelase, xylanase, and VS removal efficiency of sugarcane bagasse.

Run	Component (mL)			FPase (IU/mL)^a^		Combination effect*	CMCase (IU/mL)^a^		Combination effect*	Avicelase (IU/mL) ^a^		Combination effect*	Xylanase (IU/mL) ^a^		Combination effect*	VS removal efficiency ^a^ (%)		Combination effect*
	A:	B:	C:	Exp	Cal		Exp	Cal		Exp	Cal		Exp	Cal		Exp	Cal	
	RSC	SGS	TI															
1	0.00	0.00	3.00	2.993 ± 0.22	2.993 ± 0.22	–	0.121 ± 0.17	0.121 ± 0.12	–	0.131 ± 0.04	0.131 ± 0.04	–	0.612 ± 0.06	0.612 ± 0.05	–	55.6 ± 0.02	55.6 ± 0.01	–
2	3.00	0.00	0.00	2.788 ± 0.22	2.788 ± 0.22	–	0.061 ± 0.07	0.061 ± 0.07	–	0.059 ± 0.02	0.059 ± 0.02	–	0.521 ± 0.25	0.521 ± 0.25	–	42.3 ± 2.44	42.3 ± 1.25	–
3	0.00	3.00	0.00	2.531 ± 0.22	2.531 ± 0.22	–	0.181 ± 0.26	0.181 ± 0.25	–	0.182 ± 0.03	0.182 ± 0.02	–	0.014 ± 0.03	0.014 ± 0.02	–	42.1 ± 0.67	42.1 ± 0.52	–
4	0.00	1.50	1.50	2.718 ± 0.22	1.381 ± 0.22	1.97 ± 0.05	0.242 ± 0.07	0.076 ± 0.07	3.21 ± 0.05	0.221 ± 0.01	0.078 ± 0.02	2.82 ± 0.03	0.479 ± 0.55	0.157 ± 0.50	3.06 ± 0.01	51.6 ± 0.79	24.4 ± 0.22	2.11 ± 0.25
5	1.50	1.50	0.00	2.635 ± 0.22	1.330 ± 0.22	1.98 ± 0.01	0.173 ± 0.04	0.061 ± 0.02	2.86 ± 0.14	0.164 ± 0.02	0.060 ± 0.02	2.72 ± 0.22	0.014 ± 0.05	0.134 ± 0.02	0.10 ± 0.12	40.2 ± 1.60	21.1 ± 0.50	1.91 ± 0.02
6	1.50	0.00	1.50	0.323 ± 0.22	1.445 ± 0.22	0.22 ± 0.05	0.043 ± 0.02	0.046 ± 0.12	0.95 ± 0.04	0.041 ± 0.01	0.048 ± 0.02	0.86 ± 0.20	0.049 ± 0.03	0.283 ± 0.02	0.17 ± 0.01	40.2 ± 0.16	24.5 ± 0.22	1.64 ± 0.01
7	2.00	0.50	0.50	2.058 ± 0.22	1.390 ± 0.22	1.48 ± 0.02	0.121 ± 0.06	0.046 ± 0.02	2.66 ± 0.15	0.123 ± 0.01	0.046 ± 0.02	2.69 ± 0.02	0.028 ± 0.02	0.226 ± 0.01	0.12 ± 0.01	41.6 ± 0.16	22.2 ± 1.50	1.87 ± 0.02
8	0.50	0.50	2.00	0.988 ± 0.22	1.441 ± 0.22	0.69 ± 0.02	0.058 ± 0.09	0.061 ± 0.20	0.96 ± 0.05	0.153 ± 0.01	0.064 ± 0.01	2.40 ± 0.14	0.465 ± 0.02	0.249 ± 0.01	1.87 ± 0.22	56.2 ± 0.07	25.6 ± 0.07	2.20 ± 0.15
9	0.50	2.00	0.50	2.248 ± 0.11	1.325 ± 0.12	1.70 ± 0.01	0.184 ± 0.03	0.076 ± 0.02	2.44 ± 0.01	0.176 ± 0.03	0.077 ± 0.02	2.30 ± 0.12	0.021 ± 0.01	0.099 ± 0.02	0.21 ± 0.22	42.1 ± 0.21	22.2 ± 0.20	1.90 ± 0.12
10	1.25	1.25	0.50	2.606 ± 0.22	1.358 ± 0.22	1.92 ± 0.02	0.241 ± 0.04	0.061 ± 0.02	3.98 ± 0.02	0.251 ± 0.04	0.061 ± 0.02	4.11 ± 0.01	0.437 ± 0.03	0.162 ± 0.02	2.69 ± 0.01	50.3 ± 0.22	22.2 ± 0.20	2.26 ± 0.02
11	1.00	1.00	1.00	2.806 ± 0.01	1.385 ± 0.01	2.03 ± 0.04	0.262 ± 0.23	0.061 ± 0.22	4.33 ± 0.15	0.259 ± 0.26	0.062 ± 0.22	4.18 ± 0.01	0.599 ± 0.04	0.191 ± 0.23	3.13 ± 0.15	56.7 ± 0.63	23.3 ± 0.50	2.43 ± 0.25

*Cal.*  calculation, *Exp.*  experimental.

*Co-digestion effect > 1 indicates a synergistic effect.

*Co-digestion effect = 1 indicates no effect.

*Co-digestion effect < 1 indicates an antagonistic effect.

^a^The experimental enzyme activity or VS removal efficiency represents the total amount of enzyme activity or VS removal efficiency generated in each proportion from the cultivation process, and the calculated enzyme activity or VS removal efficiency was calculated from the enzyme activity or VS removal efficiency of the individual substrates in each microorganism source (based on mL) contained in the proportions.

### Changes in SB compositions and metabolites in the hydrolysate during KKU-MC1 cultivation

**Figure 2:**
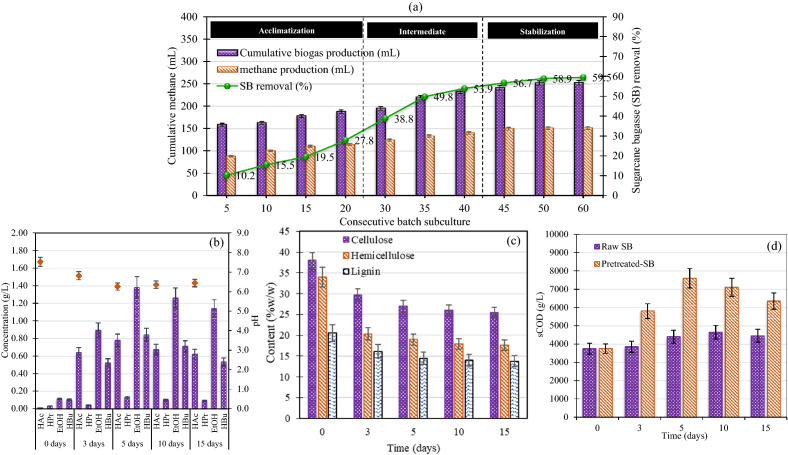
Changes in sugarcane bagasse (SB) compositions and metabolites in the hydrolysate during KKU-MC1 cultivation; methane production and volatile solid removal efficiency (**a**), volatile organic products (**b**), cellulose, hemicellulose, and lignin of the SB (**c**), and soluble COD (sCOD) (**d**). *HAc* acetic acid, *HBu* butyric acid, *HPr* propionic acid, *EtOH*; ethanol.

To confirm that Run 11 (named KKU-MC1) can be reproducible, the results on SB removal and biogas production in three stages, including acclimatization, intermediate, and stabilization, are illustrated in Fig. 2a. SB was gradually decomposed by KKU-MC1 throughout the first phase of consecutive batch subculture, which occurred from day 5 to 20. Then, the production of biogas and VS removal efficiency from SB was slightly increased. It appears that KKU-MC1 needs time to adjust to the new substrate. Then, 30–40 days into the intermediate phase, KKU-MC1 greatly increased VS removal efficiency and biogas production. At the end of the intermediate phase (day 40), VS removal efficiency, biogas production, and methane production were 19.3, 18.6, and 48.4% higher than at 20 days. The stabilization was noticed during days 45–60. During the stable period, there was less than a 2% variation in VS removal efficiency, biogas production, and methane production. On day 60, the SB removal, biogas production, and hydrogen were 59.5%, 253.1, and 151.4 mL, respectively (Fig. 2a). After the KKU-MC1 showed stable efficiency, the changes in the SB compositions and metabolites of the hydrolysate during KKU-MC1 cultivation were determined. Changes in lignocellulosic content, soluble chemical oxygen demand (sCOD), volatile organic products (VOPs), and pH during SB degradation by KKU-MC1 were investigated (Fig. 2). From Fig. 2b, the concentration of volatile fatty acids (VFAs) generally raised progressively throughout the biodegradation process and dropped after the first week until the end of the pretreatment. VFAs maximum production was assessed on the fifth day as 3.12 ± 0.3 g/L comprised of main acetic acid (HAc), and butyric acid (HBu) at pH 6.22 -6.43 through the biodegradation of KKU-MC1 (Fig. 2b). One of the key factors influencing the medium pH value and the efficiency of the methane production process is VFA concentration^28^. The optimal pH range for producing VFAs was between 5.3 and 11 has been reported^29^. In this study, the pH ranges for the pretreatment of SB with KKU-MC1 that resulted in the highest production of VFAs were 6.2 to 6.4. Therefore, the pH value of 6.8 to 7.2 during the pretreatment process is an efficient parameter in enhancing the methane production process^30^. Differences in weight loss and the breakdown of lignocellulosic biomass may cause pH changes. The changes in weight loss in terms of cellulose, hemicellulose, and lignin during the 15 days of KKU-MC1 cultivation are shown in Fig. 2c. Over 5 days, KKU-MC1 could break down cellulose, hemicellulose, and Lignin from the SB with efficiencies of 30.6 ± 3.2, 47.2 ± 2.5, and 29.2 ± 5.1%, respectively, consistent with the VS removal efficiency of 56.7%. The performance of MC on lignocellulose degradation has been reported by Ali et al. ^31^, who used BC-4 enriched from milled sawdust for pretreating the catalpa sawdust for 15 days at 50 °C, that capable of degrading cellulose, hemicellulose, and lignin 47.5, 58.7 and 40.3%, respectively. However, this depends on the substrate's complexity and the process's temperature. Results revealed that the pre-hydrolyzed SB with KKU-MC1 is an effective pretreatment capable of breaking the link between polysaccharide and lignin. Thus, anaerobic bacteria are more accessible and easier to digest cellulose and hemicellulose. As a result, sugar monomers (in this study, we investigated the sCOD that is released during KKU-MC1 treated SB) and VFAs were produced, which can be bio-converted for biogas production^32^. Regarding the sCOD alterations, KKU-MC1 significantly raised its concentration in the hydrolysate during the early stages of degradation (Fig. 2d). On the fifth day, the maximum sCOD concentrations with KKU-MC1 were seen, measuring 7610 ± 50.2 mg/L, respectively. After that, a drop in sCOD concentrations was observed until the 15th day. The pretreated SB with KKU-MC1 has a high sCOD, indicating that it is highly biodegradable, enhancing the sCOD up to 42.1% compared with the control at 5 days. The increased sCOD found in this study was comparable to that of Choudhury et al. ^33^. (7,680 mg/L), who biologically pretreated petroleum refinery sludge with *Pseudomonas* *putida*. Additionally, Merrylin et al. (2013), who used *Bacillus* *licheniformis* strains to biotreat urban wastewater sludge, had sCOD values 2.4 times lower than our work^34^. Through the process, the untreated SB (control) showed a low concentration of sCOD in the range of 3850–4450 mg/L. Only a few changes in sCOD were noticed (Fig. 2d). It is evident that during KKU-MC1 cultivation, the concentration of VFAs increased, the pH dropped, and the level of sCOD increased until the fifth day. A subsequent drop in VFAs and sCOD concentrations and increased pH values were observed reaching the 15th day. Poszytek et al. ^35^ reported that the optimal pretreatment period should correspond with the highest VFAs and sCOD concentrations. In our investigation, this value was attained within 5 days of biodegradation. However, the subsequent decline can be caused by the microbial consortia's anabolic and catabolic processes. Thus, the subsequent reduction may be due to the anabolic and catabolic processes of the MC^12^. Results indicated that KKU-MC1 has high extracellular endo- and exoglucanase, which can decompose complex molecules of lignocellulose materials with high sCOD release VFAs that can be bio-converted for biogas production.

### Taxonomic and functional profiles of predicted genes in the metagenome

**Figure 3:**
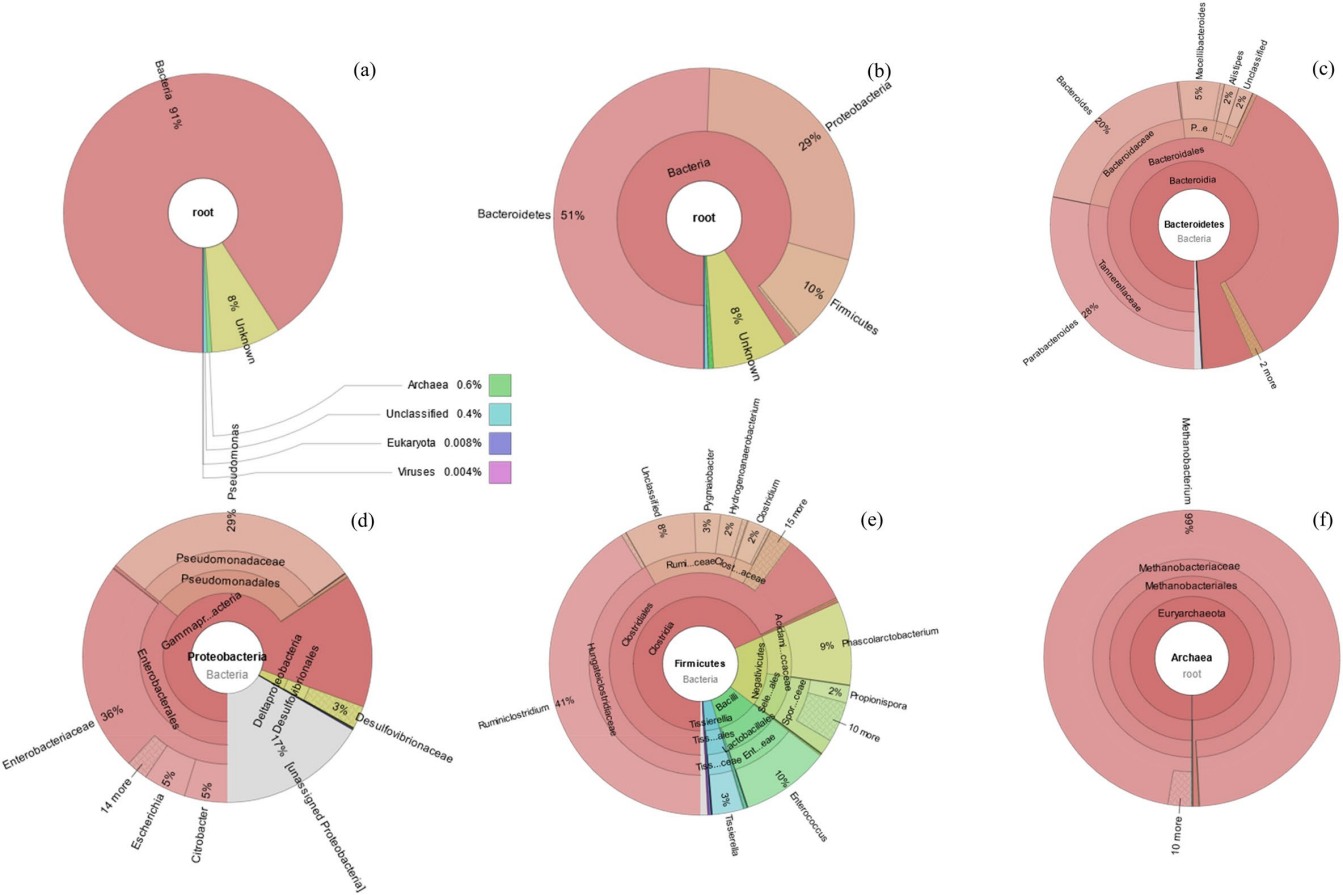
Abundance taxonomy of the KKU-MC1 at Kingdom level (**a**), phylum level (**b**), genus level of phylum *Bacteroides* (**c**), genus level of phylum *Proteobacteria* (**d**), genus level of phylum *Firmicutes* (**e**), and at genus level of Archaea (**f**).

Metagenome sequencing of KKU-MC1 generated a total of 7.69 Mbp high-quality reads (Table 4). After de novo assembly, 36,606 scaftigs longer than 500 bp were obtained. The metagenome of KKU-MC1 was predicted to contain 85,894 open reading frames, with an average length of 700.31 bp. Taxonomic classification revealed that KKU-MC1 was primarily composed of bacteria (91%) and archaea (0.6%). The rests were Eukaryota and unknown (Fig. 3a). Phylum *Bacteroidetes* (51%) was found to be the minority population in MC, followed by *Proteobacteria* (29%) and *Firmicutes* (10%) (Fig. 3b). Other phyla such as *Spirochaetes,* and *Actinobacteria*, were present at low abundances. Compared to other published lignocellulosic metagenomes, we noted that the community structure in KKU-MC1 was similar to a microbial community inhabiting a biogas reactor^36^ and MC^7,8^. The MC was enriched from compost habitats and adapted to grow on untreated corn stover and was found to be predominantly composed of members of the phylum *Proteobacteria*, followed by *Bacteroidetes* and *Firmicutes*. Kanokratana et al. and Zhu et al. reported that the MC was constructed from forest compost soil microbiota on NG^7,8^. There were also abundant *Proteobacteria, Firmicutes*, and *Bacteroidetes*. Mhuantong et al. found that the phylum *Proteobacteria* was abundant in SB, where the biomass is actively degraded by lignocellulolytic microbiota and has a variety of endo-acting enzymes that can degrade cellulose, hemicellulose, and lignin^37^. KKU-MC1 originates from sources involved in cellulose degradation (including RSC, SGS, and TI), with SB as a carbon source, which is consistent with published data. Previous research has reported that *Firmicutes* and *Bacteroidetes* are found predominantly in environments involved in the degradation of cellulose, such as compost heaps^38^, the gut of termites^39^, the thermophilic composting phase, and sheep microbiome^40^. In the SB, *Proteobacteria* were found to be especially abundant, followed by *Firmicutes*^41^.

**Table 4:** Summary results of cellulolytic consortium KKU-MC1 metagenome.

Parameters sequencing results	Parameters sequencing results
Total raw data (Mbp)	7.69
Assembly and mix-assembly	
Scaffolds (average)	36,606
Total length (bp)	141,972,579
Average length (nt)	1,939
Longest length (nt)	655,703
N50 (bp)	4,055
N90 (bp)	632
Gene prediction	
Total ORFs	171,787
Average ORFs	85,894
Average length (bp)	700.31
GC percent (%)	47.93
Taxonomic annotation	
Gene catalogue	139,891
Annotated on NR	92,787 (66.33%)
Annotated on unclassified	4.56%
Annotated on kingdom level	95.44%
Annotated on phylum level	94.29%
Annotated on class level	91.76%
Functional annotation	
Gene catalogue	139,891

Focusing on a more refined taxonomic level (Fig. 3c), the genus *Parabacteroides* (28%), followed by *Bacteroides* (20%)*, Macellibacteroides* (5%), and *Alistipes* (2%), were the most abundant in the phylum *Bacteroidetes*. Previous studies have shown a positive correlation between biomass hydrolysis and the population of *Parabacteroids*^42^. Strictly anaerobic cellulolytic bacteria of the genus *Bacteroides* can ferment various sugars (e.g., glucose, sucrose, fructose, maltose, xylose, galactose) and pectin, starch, and cellulose to HAc and HSc (succinic acid)^43^, facilitating biogas production. Moreover, Amin et al. reported that acetogenic bacteria that produce hydrogen and acetate, *Macellibacteroides*, are capable of metabolizing monosaccharides and disaccharides to produce HBu, HAc, and iso-butyrate (iso-HBu)^44^. According to Won et al., *Alistipes* can degrade lignocellulose and produce VFAs ^45^. The second most abundant is phylum *Proteobacteria* with the dominant genera *Pseudomonas* (29%) and *Citrobacter* (5%) (Fig. 3d). Both bacteria genera are known as extracellular enzyme producers^46^. In addition, *Pseudomonas* have been studied for their ability to break down lignin to synthesize bio-products^47^. The third largest abundance analyzed was allocated to the phylum *Firmicutes*, with the dominant genera *Ruminiclostridium* (41%), *Enterococcus* (10%) *Phascolarctobacterium* (9%), and *Tissierella* (3%) (Fig. 3e). Among them, *Ruminiclostridium* is responsible for cellulose and hemicellulose degradation and hydrogen production^48^. Moreover, the MC named TC-5 was mainly composed of *Ruminiclostridium* (63%), showed a variety of lignocellulolytic enzyme activity, and increased methane production by 22–36% during AD of untreated wheat straw^10^. Furthermore, *Enterococcus* has been associated with lignocellulosic waste fermentation^49^. It is well known that the primary metabolic pathway in *Enterococcus* can be changed based on its growth conditions, with the primary end fermentation producing VOPs depending on the carbon source^50^. According to previous research, *Tissierella* was also involved in HAc and HBu production^51^. *Phascolarctobacterium* could metabolize different substrates, monosaccharides, disaccharides, proteins, and HSc, into propionic acid (HPr) and HBu^52^. KKU-MC1 also contained the methanogenic archaea, classified as members of the genus *Methanobacterium* belonging to the phylum *Euryarchaeota* (Fig. 3f). The hydrogenotrophic, *Methanobacterium* is one of the methanogens producing methane from hemicellulose, which possess the same methanogenesis pathway. Moreover, this genus is aciduric and can grow at low pH conditions (< 6.0)^53,54^. In addition, the high-throughput microbial analysis indicated that *Methanobacterium* is one of the members that significantly contributed to the high MY from the saccharification-expanded corn straw treated with *Clostridium* *thermocellum* XF811^55^. According to the findings, utilizing the KKU-MC1 characteristics of bacteria and methanogens is attractive for in situ bioaugmentation in the AD of lignocellulosic biomass.

**Figure 4:**
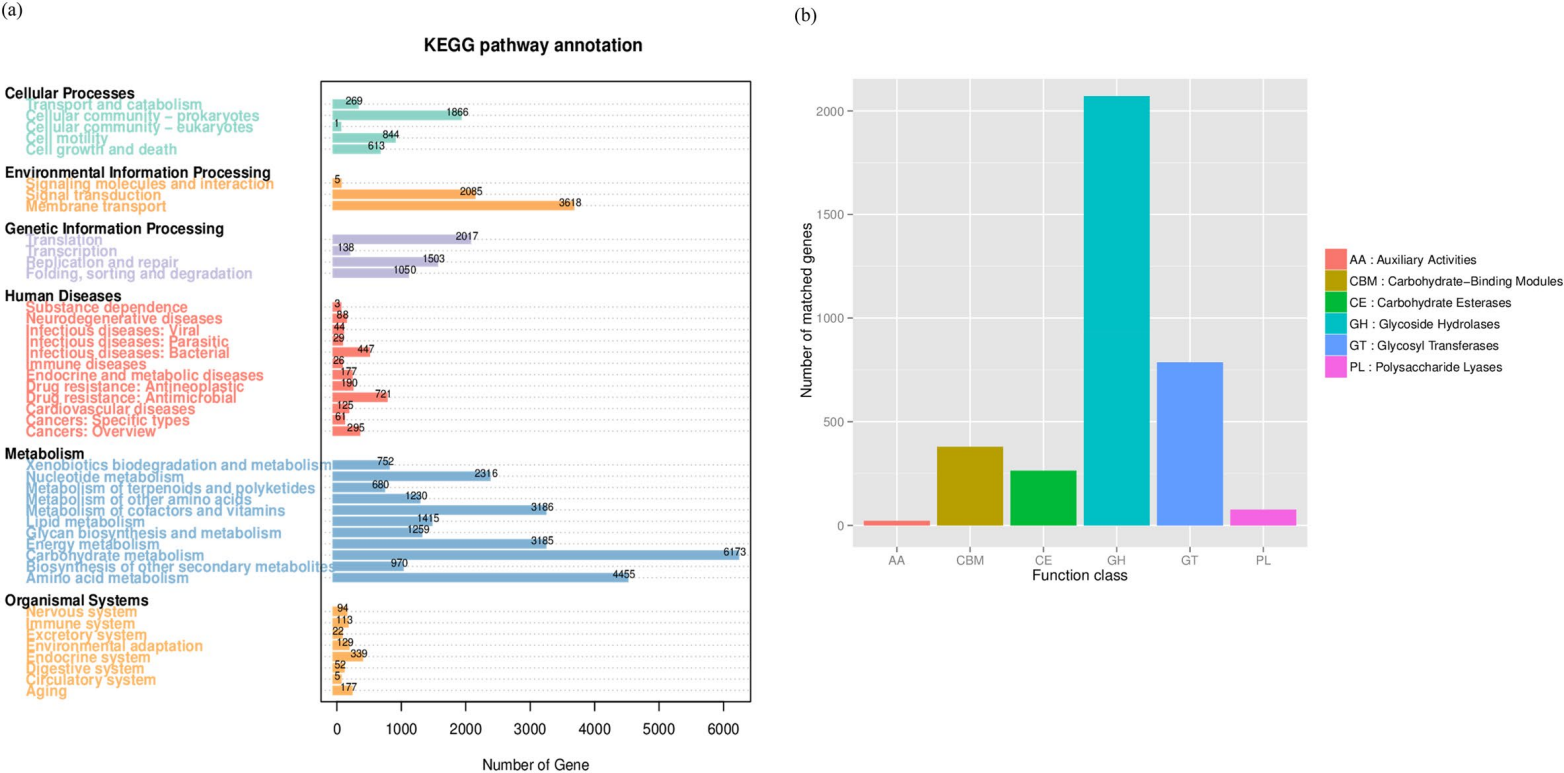
Functional genes annotated in the KEEG database (Level 2) (**a**) and annotated in the CAZy database (**b**) showing the number of genes (Level 1) of the cellulolytic consortium KKU-MC1.

To understand the functions and metabolic pathways of the microorganisms in KKU-MC1, the predicted proteins from the metagenomic data set were analyzed for their predominant functions using the Kyoto Encyclopedia of Genes and Genomes (KEGG) (Fig. 4a), evolutionary genealogy of genes: Non-supervised Orthologous Groups (eggNOG) databases. In the KEGG database (Fig. 4b), the number of genes associated with the “Metabolism” categories constituted 52.25% (25,621 genes), with “Carbohydrate metabolism” dominating at 14.43% (6,173 genes), which were the most relevant for lignocellulose degradation. The detailed analysis of the subcategories of “Carbohydrate metabolism” allows the detection of microbial enzymes involved in the degradation of lignocellulose biomass, with fructose and mannose (779 genes, 17.21% of total carbohydrate metabolism) and galactose (739 genes, 16.26% of total carbohydrate metabolism), which is an intermediate product of lignocellulose degradation (Supplementary Table S3). In addition, the categories of “Metabolism” were primarily “Amino acid metabolism” (10.43%; 4,455 genes), “Metabolism of cofactors” (7.45%; 3,186 genes), and “Vitamins and energy metabolism” (7.45%; 3,185 genes).

The second most prominent functional gene was CAZymes, consisting of 5,708 genes (16.33%) associated with “Environmental information processing,” “Membrane transport” (8.46%; 3618 genes), and “Signal transduction” (4.88%; 2085 genes) as predominant categories. The proportion of genes in the category of “Organismal system” (2.22%), and “Human diseases” (5.53%) was deficient. Metabolic-profile mapping using the eggNOG databases showed results similar to those of the KEGG orthology (KO) categories (KEGG database). Based on the functional categories of clusters of orthologous groups of proteins (COGs), approximately 29.79% (24,537 genes) of all proteins in the metagenome were poorly characterized (Supplementary Table S4). Most of the assigned functional proteins are involved in “Amino acid transport and metabolism” (8.04%; 6619 genes) and “Carbohydrate transport and metabolism” (7.85%; 6467 genes). The annotation results of the KEGG, and eggNOG databases showed that in KKU-MC1, the metabolic activities associated with carbohydrate hydrolysis were very active. This finding suggested the similarity in metabolic patterns analyzed in the metagenome of the lignocellulolytic microbial consortium EMSD5 during growth on corn stover^8^, in decayed *Torreya nucifera* (L) of Bija forest^15^, and SB and cow manure compost^14^ showed a prevalence of the functional genes related to carbohydrate degradation. It is worth noting that this study aimed to investigate the taxonomic and enzymatic basis of the cellulolytic microbial consortium KKU-MC1 and its application for enhancing biomethane production from various lignocellulosic waste in batch fermentation. Therefore, an in-depth understanding of the microorganisms and the activity of specific microbes involved in biomass degradation during the AD process and monitoring the modifications that occurred during the changes in environmental conditions using metatranscriptomics should be investigated. Additionally, the transcriptionally active members and their most abundant functions within the microbial community from up-scale biogas production should be investigated to comprehend the mechanisms of adaptation of the microbial consortium to high VS loading of lignocellulosic biomass, leading to the development of metabolic engineering approaches to overcome this challenge.

### Diversity and taxonomic affiliation of CAZyme genes

To gain insights into the carbohydrate metabolism in KKU-MC1, we screened our metagenome for genes encoding enzymes that catalyze the hydrolysis of plant polymers. The KKU-MC1 consortia contained 3599 different CAZyme genes distributed unequally between glycoside hydrolases (GH) with 2027 genes (57.57%), glycosyltransferase (GT) family with 786 genes (21.84%), carbohydrate-binding module (CBM) family with 379 genes (10.53%), carbohydrate esterase (CE) family with 264 genes (7.34%), auxiliary activity (AA) family with 22 genes (0.62%), and polysaccharide lyases (PLs) with 76 genes (2.11%) (Fig. 4b). As shown in Table 5, CAZymes, which degrade lignocellulosic substrates, include 28 different families of GHs. Among the cellulases, five families (GH 3, 5, 8, 9, 63, and 74) were most abundant in GH3 (145 genes), followed by GH9 (37 genes) and GH5 (27 genes). These GH families encompass a variety of hydrolytic enzymes, such as cellulases, endo- and exoglucanases, cellobiohydrolases, and β-/α-glucosidase. For hemicellulose degradation, 23 families were present, the most prevalent of which was GH2 (β-galactosidase) with 190 genes, GH43 (including βxylosidase; α-l-arabinofuranosidase) with 159 genes, GH92 (α-mannosidase) with 97 genes, and GH1 (β-glucosidase) with 83 genes. The downstream decomposition of hemicellulose is mainly catalyzed by various oligosaccharide-degrading enzymes such as β-glucosidase and β-xylosidase^15^, which are also present in KKU-MC1 (GH1 and GH43, respectively). Additionally, GHs targeting other polysaccharides, such as GH13 (α-amylase), GH15 (glucoamylase/ glucodextranase), GH65 (α-trehalase), as well as GH78, and GH106 (both as α-L-rhamnosidase) were identified in KKU-MC1. We also observed four AA families, redox-active enzymes that may be involved in lignin deconstruction, allowing the GH, GT, PL, and CE enzymes to act on the saccharidic polymers in the biomass. Most lignin-degrading enzymes were multicopper oxidase (AA1, 6 genes) and GMC oxidoreductase (GMC: glucose-methanol-choline; AA3, 6 genes). 1,4-benzoquinone reductase (AA6, 4 genes) and glucooligosaccharide oxidase (AA7, 2 genes). Additionally, the presence of CBM50 (168 genes) in KKU-MC1 enhanced the binding capacity of lignocellulose and enzymes (Supplementary Table S5), leading to more efficient degradation of lignocellulose materials, according to research by Zhong et al.^56^. The presence of CBM50, commonly found in microbial consortia enriched from compost^57^, anaerobic digesters fed with wastewater treatment sludge^58^, and camel rumen microbiome^59^. The extensive complement of CAZymes expressed in KKU-MC1 reflects the extraordinary ability to degrade the diversity of polysaccharides available in the complex substrate.

**Table 5:** The number of CAZymes according to lignocellulose degradation identified from the metagenome.

CAZy family	Known activities	Count
Lignin		
AA1	Multi copper oxidase	6
AA3	GMC oxidoreductase	6
AA6	1,4-Benzoquinone reductase	4
AA7	Glucooligosaccharide oxidase	2
Cellulose		
GH3	β-Glucosidase	145
GH5	Cellulase/endo-& β-1,4 glucanase/β-1,4 Cellobiosidase	27
GH8	Cellulase	10
GH9	Cellobiohydrolase/β-glucosidase/exoglucanase	37
GH63	β-Glucosidase/endoglucanase	10
GH74	Endoglucanase/xyloglucanase	2
Hemicellulose		
GH1	β-Glucosidase	83
GH2	β-Galactosidase	190
GH4	α-Glucosidase; α-galactosidase	34
GH10	Endo-1,4-β-xylanase; endo-1,3-β-xylanase	16
GH16	Galactosidase	17
GH26	β-Mannanase	17
GH27	α-Galactosidase	11
GH29	α-l-fucosidase	37
GH30	Endo-β-1,4-xylanase; β-glucosidase	32
GH31	α-Glucosidase; α-1,3-glucosidase	26
GH35	Exo-β-glucosaminidase	15
GH36	α-Galactosidase	29
GH38	α-Mannosidase	16
GH42	β-Galactosidase; α-l-arabinopyranosidase	10
GH43	β-Xylosidase; α-l-arabinofuranosidase	159
GH51	α-l-arabinofuranosidase/endoglucanase	29
GH57	α-Galactosidase	13
GH92	α-Mannosidase	97
GH95	α-1,2-l-fucosidase	30
GH97	α-Glucosidase; α-galactosidase	90
GH115	α-Glucuronidase; alpha-1,2-glucuronidase	24
GH125	Exo-α-1,6-mannosidase	14
GH127	β-l-arabinofuranosidase	19
Other oligosaccharide-degrading enzymes		
GH13	Amylase	116
GH15	Glucoamylase; glucodextranase	8
GH65	α-Trehalase	14
GH78	α-l-rhamnosidase	52
GH106	α-l-rhamnosidase	17

**Figure 5:**
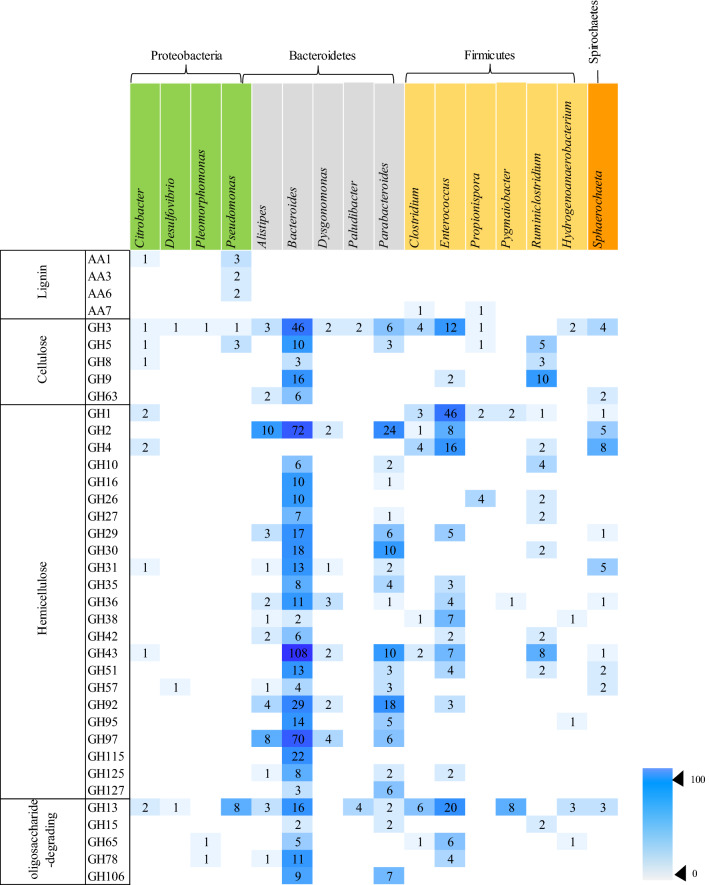
Heat map showing the distribution of glycoside hydrolase (GH), and auxiliary activities (AA) families in the abundant genera. CAZy families are grouped according to their activities on major components of plant cell walls. Only GH families targeting (hemicellulose and cellulose), and AA families targeting lignin are considered. Only genera with ten or more GHs are presented. *Proteobacteria* are depicted in green, *Bacteroidetes* in gray, *Firmicutes* in yellow, and S*pirochaetes* in orange.

**Figure 6:**
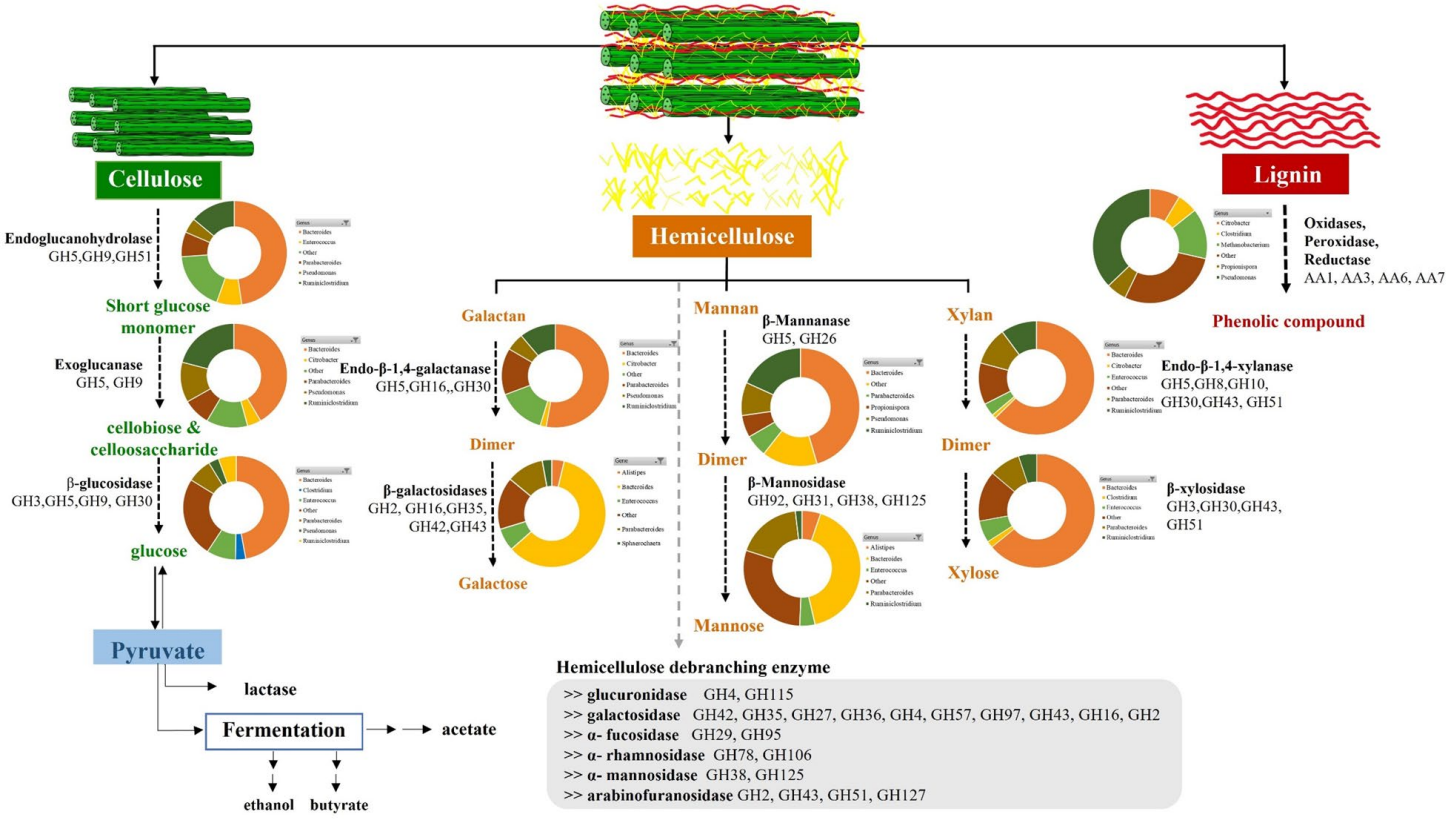
Lignocellulosic degradation pathway, related enzymes, and microorganisms found in sugarcane bagasse metagenome. Colored pie charts show the number of major bacterial phyla microorganisms involving different steps of biomass degradation. (I) Cellulose (green): Full enzymatic hydrolysis of cellulose requires the cooperation of endoglucanase [3 glycoside hydrolase (GHs)], exoglucanase (2 GHs), and β-glucosidase (4 GHs); (II) Hemicellulose main chain (orange): degradation of the hemicellulose linear β-1,4-linked main chain requires the action of endo-β-1,4-xylanase (6 GHs) and β-xylosidase (4 GHs) for β-1,4-xylan, and β-mannanase (2 GHs) for β-1,4-mannan, and β-galactanase (3) for β-galactosidase. (III) debranching enzymes (gray): debranching enzymes for degradation of complex substituted xylan included ten GHs galactosidase, two GHs α-fucosidase, two GHs α-rhamnosidase, two GHs α-mannosidase, four GHs arabinofuranosidase and debranching enzymes for pectin included two GHs glucuronidase. (IV) lignin (red): KKU-MC1 exhibited with lignin degradation activity with four AAs; AA1, AA3, AA6, and AA7.

The distribution of lignocellulolytic CAZymes with the most abundant genus found in the KKU-MC1 metagenome is shown in Fig. 5. Shades of dark blue to light blue represent low and high numbers of CAZymes, respectively, found in each bacterial genus. The taxonomic distribution of GHs showed that 6 of 16 genera with the most GHs in KKU-MC1 were from *Firmicutes*, compared to 4, 5, and 1 from *Proteobacteria*, *Bacteroidetes*, and *Spirochaetes*, respectively. Our results show that four types of bacterial phyla encode cellulases that degrade exo- and oligosaccharides in different amounts (GH 3, 5, 8, and 9), and *Bacteroidetes* bacteria were predicted to produce α-glucosidase and α-1,3-glucosidase (GH 63). The number of genes encoding the cellulolytic GH enzyme linked to *Bacteroidetes* was 11, 3, and 16 times higher than that of *Firmicutes*, *Proteobacteria*, and *Spirochaetes*, respectively Fig. 5. Most of the putative enzymes belonging to cellulolytic GH families 3, 5, 8, 9, and 63 were found in members of *Bacteroidetes*, especially *Bacteroides*. It was also found in members of *Firmicutes*, especially *Ruminiclostridium* and *Enterococcus*. CAZyme, which is involved in hemicellulose degradation, is distributed across four phyla. The highest hemicellulolytic GH genes were also observed in the genera *Bacteroides* and *Parabacteroides*, which belong to the phylum *Bacteroidetes* Fig. 5. It was also found in the phylum *Firmicutes*, especially *Enterococcus*. However, the number of genes encoding the hemicellulolytic GH enzyme linked to Phylum *Proteobacteria* was the lowest, and only six genes originated from the genus *Citrobacter*. In terms of lignin degradation, both *Firmicutes* and *Proteobacteria* possessed 18 genes encoding AA family enzymes. However, only two genes of AA2 were assigned to *Pseudomonas* members of the phylum *Proteobacteria* Fig. 5. These observations suggest that microbial populations may have different metabolic niches within the consortium during the decomposition of lignocellulosic biomass. Members of *Bacteroidetes,* and *Firmicutes* harbored an enriched catalog of genes encoding potential cellulases, and hemicellulases for degrading complex polysaccharides such as cellulose, and hemicellulose. Meanwhile, *Proteobacteria* was richer in gene families involved in lignin degradation. Different expression patterns of enzyme arrays that digest complex lignocellulose biomass indicate that KKU-MC1 can be used to digest a variety of fiber substrates into biogas. These CAZymes are summarized along with their mechanism profiles of lignocellulose degradation and the predominant microorganisms responsible for each degradation step in Fig. 6. It was demonstrated that KKU-MC1 contained almost the complete set of enzymes required to degrade cellulose and hemicellulose.

### Methane production from lignocellulosic material using KKU-MC1

#### Bioaugmentation with KKU-MC1

**Figure 7:**
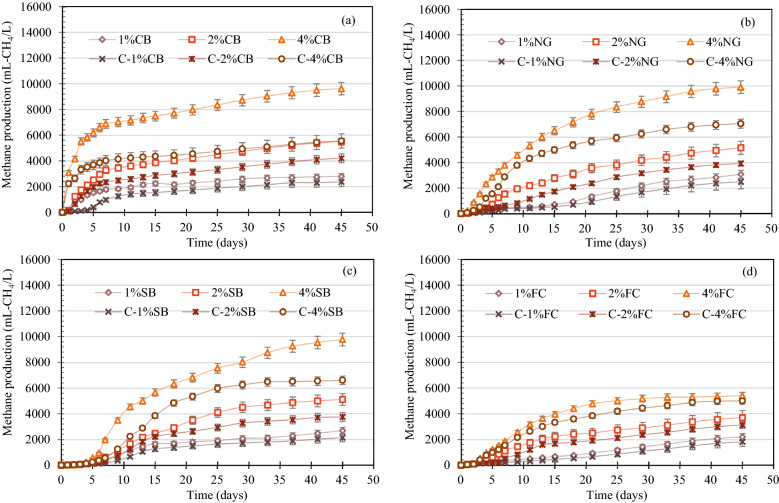
Methane production from bioaugmentation treatment at 1, 2, and 4% initial volatile solid concentration (IVC) of each substrate, including cassava bagasse (CB) (**a**), Napier grass (NG) (**b**), sugarcane bagasse (SB) (**c**), and filter cake (FC) (**d**). C (control) indicates the non-bioaugmentation treatment at various %IVC of each substrate.

The effect of bioaugmentation with 10% v/v KKU-MC1, rich in *Bacteroides* and *Proteobacteria*, was elucidated under varying IVC of each substrate and compared to controls (non-augmentation; Control (-C)) in AD batch experiments. The methane production at 1, 2, and 4% IVS of each substrate CB, NG, SB, and FC augmented with KKU-MC1 was higher than the control of each substrate and continued to increase after a short lag phase and peaked on day 45 (Fig. 7). The methane production of CB at 1, 2, and 4% IVC augmented with KKU-MC1 at day 3 higher than non-augmentation by 87.9, 38.8, and 39.8%, respectively. On day 5, the methane production of NG and SB at 1, 2, and 4% IVC augmented with KKU-MC1 was 38–44, 26–43, and 46–59% higher than non-augmentation of NG and SB at 1, 2, and 4% IVC, respectively. Likewise, the methane production of FC augmented with KKU-MC1 on 7 days was higher than non-augmentation by 18–43% at all IVC. It was illustrated that KKU-MC1 could quickly boost the speed of lignocellulosic degradation and methane production compared with non-augmentation. This phenomenon could be due to AD augmented by KKU-MC1 that encode lignocellulolytic enzymes, that is, cellulases, hemicellulases, and lignolytic enzymes, which can support biomass depolymerization, increase rates of hydrolysis and, in turn, methane production, while also promoting digester microbial persistence according to Mulat et al. and Ozbayram et al.^60,61^. At the end of the AD of augmentation treatment, the highest methane production of 9620 ± 105.5, 9907 ± 120.5, 9780 ± 94.0, and 5400 ± 114.0 mL-CH_4_/L were achieved at 4% IVC of the CB, NG, SB, and FC respectively, which increased by 73, 33, 37, and 8%, respectively, compared to the control of each substrate (Fig. 7a–d). At 2% IVC, the methane production of the CB, NG, SB, and FC augmented by KKU-MC1 increased by 31, 32, 36, and 18%, respectively, compared to the controls. In addition, a slight increase in methane production of the CB, NG, SB, and FC was augmented by KKU-MC1 in the range of 18–25% compared to the control of each substrate, which was observed at 1% IVC. However, at all IVCs, there was no significant difference in the lag phase period of methane production from the CB and C-CB (Table 6). Meanwhile, the lag phase period of methane production of NG, SB, and FC augmented with KKU-MC1 at all IVCs was significantly shorter than those of non-augmentation NG, SB, and FC at all IVCs (Table 6). The high methane production and short lag phase time obtained from augmentation KKU-MC1 could be due to the low lignin content in each material (Fig. 2b). Lignin serves as a physical barrier that prevents cellulase enzymes from accessing the cellulose component, limiting the effectiveness of hydrolysis. Bioaugmentation is a direct method to increase the performance of fermentation by using microorganisms with biodegradability. The effectiveness of the polymer hydrolysis, which is the AD rate-limiting phase, has been carried out with hydrolytic bacteria by enhancing the conversion of recalcitrant lignocellulose with positive outcomes ^62^.

**Table 6:** Methane production of lignocellulosic material augmentation with 10%v/v KKU-MC1, and non-augmentation (control: C-).

Substrate	% IVC	MY (mL-CH_4_/g-VS_added_)	Calculated values from Gompertz model			First order model	
			MPR (mL-CH_4_/ g-VS·day)	*k*_*h*_ (day^−1^)	R^2^	Lag phase (day)	R^2^
CB	1	280.2 ± 75.5	11.7 ± 3.5	0.151 ± 0.008	0.972	1.3 ± 0.5	0.927
	2	275.4 ± 25.0	11.5 ± 3.0	0.134 ± 0.005	0.973	1.4 ± 1.1	0.926
	4	240.5 ± 93.0	10.0 ± 2.5	0.168 ± 0.015	0.934	1.6 ± 0.3	0.963
C-CB	1	237.1 ± 20.0	9.9 ± 1.0	0.073 ± 0.003	0.985	1.3 ± 0.6	0.951
	2	210.3 ± 12.0	8.8 ± 2.2	0.132 ± 0.010	0.970	1.5 ± 0.9	0.908
	4	138.9 ± 33.0	5.8 ± 3.0	0.164 ± 0.004	0.937	1.6 ± 0.2	0.984
NG	1	308.7 ± 23.0	12.9 ± 2.0	0.037 ± 0.007	0.989	1.3 ± 0.3	0.981
	2	258.1 ± 55.0	10.8 ± 1.5	0.059 ± 0.012	0.972	1.9 ± 0.2	0.984
	4	247.7 ± 30.0	10.3 ± 2.0	0.081 ± 0.004	0.999	5.1 ± 0.4	0.991
C-NG	1	249.1 ± 22.0	10.4 ± 2.0	0.024 ± 0.007	0.959	2.5 ± 0.1	0.989
	2	196.2 ± 20.0	8.2 ± 1.5	0.027 ± 0.003	0.978	3.0 ± 0.5	0.989
	4	176.3 ± 12.5	7.3 ± 3.0	0.041 ± 0.014	0.978	7.0 ± 1.3	0.991
SB	1	269.0 ± 29.0	11.2 ± 1.5	0.036 ± 0.014	0.965	3.4 ± 0.2	0.995
	2	256.0 ± 40.1	10.7 ± 1.0	0.055 ± 0.004	0.967	3.8 ± 0.2	0.994
	4	244.5 ± 22.4	10.2 ± 2.2	0.085 ± 0.005	0.964	3.9 ± 0.1	0.992
C-SB	1	214.5 ± 16.5	8.9 ± 0.8	0.012 ± 0.005	0.949	5.3 ± 0.2	0.992
	2	188.0 ± 9.5	7.8 ± 0.9	0.012 ± 0.008	0.950	5.2 ± 1.4	0.991
	4	165.3 ± 13.0	6.9 ± 3.0	0.012 ± 0.004	0.948	6.2 ± 1.1	0.993
FC	1	220.5 ± 20.5	9.2 ± 0.5	0.025 ± 0.004	0.998	1.8 ± 0.5	0.998
	2	185.1 ± 15.5	7.7 ± 3.0	0.054 ± 0.009	0.998	2.9 ± 1.1	0.998
	4	135.0 ± 20.0	5.6 ± 0.8	0.070 ± 0.011	0.998	3.7 ± 1.2	0.999
C-FC	1	182.0 ± 25.0	7.6 ± 3.0	0.010 ± 0.006	0.991	3.0 ± 1.0	0.992
	2	156.3 ± 10.0	6.5 ± 0.9	0.012 ± 0.004	0.988	4.1 ± 1.0	0.999
	4	125.1 ± 11.0	5.2 ± 1.0	0.010 ± 0.005	0.994	7.4 ± 1.4	0.991

The maximum methane production rate (MPR) was observed in the KKU-MC1-augmentation of the CB, NG, SB, and FC at all IVC (Table 6). The MPR of the CB at 1, 2, and 4% IVC augmented by KKU-MC1 showed 11.7 ± 3.5, 11.5 ± 3.0, and 10.0 ± 2.5 mL-CH_4_/g-VS·d, respectively, which were higher than the control by 15–42. Additionally, the MPR of the NG at 1, 2, and 4% IVC augmented with KKU-MC1 had 12.9 ± 2.0, 10.8 ± 1.5, and 10.3 ± 2.0 mL-CH_4_/g-VS·d, respectively, which were higher than the control by 19–29%, respectively. The MPR of the SB at 1, 2, and 4% IVC augmented by KKU-MC1 presented 11.2 ± 1.5, 10.7 ± 1.0, and 10.2 ± 2.2 mL-CH_4_/g-VS·d, respectively, which increase in the range 20–32% compared to the control. Contrastingly, the MPR of the FC augmented by KKU-MC1 tended to decrease with increasing IVC of the substrate. The MPR of the FC at 1, 2, and 4% IVC augmented with KKU-MC1 were 9.2 ± 0.5, 7.7 ± 3.0, and 5.6 ± 0.8 mL-CH_4_/g-VS·d, respectively, which were higher than the control by 7–17%. The results showed that the loading of 10% v/v of KKU-MC1 into the AD process increased the MPR of the CB, NG, SB, and FC despite high IVC. The MPR results indicated that KKU-MC1 could improve the MPR in the order CB > SB > NG > FC, depending on the lignocellulosic content of each substrate. The CB has a lower lignin content (12.61 ± 5.6%) than the NG, and SB (see Table 1), so lignin and hemicellulose are readily degraded, and microbes use cellulose more easily. Meanwhile, the lowest MPR of FC could be due to an imbalance of the carbon-to-nitrogen (C:N) ratio in the AD process. The FC had a relatively low C:N ratio (24:1), around the lowest recommended limit^63^. Thus, a co-digestion strategy was introduced to balance C:N nutrients to improve this substrate's degradability and energy production.

We attempted to understand how adding KKU-MC1 to the AD process optimizes the degradation of lignocellulose to fermentation products. Therefore, when no microbial inhibition occurs during the AD process, it can be assumed that the overall process rate is primarily driven by hydrolysis, which can be explained by the hydrolysis rate constant (*k*_*h*_). Our results showed that the AD process augmented with KKU-MC1 showed a faster hydrolysis rate. The *k*_*h*_ of the CB at 1, 2, and 4% IVC augmented by KKU-MC1 was 0.151, 0.134, and 0.168 day^−1^, respectively, while the control of the CB at 1, 2, and 4% IVC was lower (0.073, 0.132, and 0.164 day^−1^, respectively) (Table 6). In comparison, for the NG augmented with KKU-MC1, the *k*_*h*_ value increased with increasing IVC, which was 0.037, 0.059, and 0.061 day^−1^, while the control of the NG had lower *k*_*h*_ values (0.024, 0.027, and 0.041 day^−1^). In addition, *k*_*h*_ values increased with increasing IVC for the SB, and FC augmented with KKU-MC1. SB and FC augmented with KKU-MC1 (at 1, 2, and 4% IVC) showed *k*_*h*_ values of 0.036, 0.055, and 0.085 day^−1^, respectively, and 0.025, 0.054, and 0.070 day^−1^, respectively. The SB and FC control had *k*_*h*_ values of approximately 0.012 day^−1^ for all IVC. Adding KKU-MC1 to the AD system increased the *k*_*h*_ values of all substrates. The augmentation with KKU-MC1 to the CB at 1% IVC improved *k*_*h*_ (2 times) compared to the control. However, augmentation with KKU-MC1 in the NG, SB, and FC at high IVC (2 and 4%) improved the *k*_*h*_ values by 2 to 7 times compared to the control of the respective substrates. This may be due to the recalcitrant lignocellulose structure of the non-augmented substrate, which is difficult for hydrolytic bacteria to absorb and utilize, and therefore take a long time to hydrolyze, resulting in a lower *k*_*h*_ value. Non-augmentation treatments were inferior to hydrolytic bacteria compared to augmentation treatments, resulting in inadequate degradation of raw CB, NG, SB, and FC. This causes a reduction in simple sugars. As indicated in Table 6, low quantities of simple sugars result in less VFAs converted to acetate, resulting in a smaller MY than that induced by KKU-MC1.

The MY of the CB at 1, 2, and 4% IVC augmented by KKU-MC1 were in the range of 241–280 mL-CH_4_/g-VS_added_, which increased by 15.4, 24.6, and 42.2% compared to the control, respectively (Table 6). In addition, the MY of the NG, and SB augmented with KKU-MC1 at 1, 2, and 4% IVC ranged between 245 and 309 mL-CH_4_/g-VS_added_, which increased by 19–32% compared with the control, respectively. The MY of the FC augmented with KKU-MC1 at 1, 2, and 4% IVC was 220.5 ± 20.5, 185.1 ± 15.5, and 135.0 ± 20.0 mL-CH_4_/g-VS_added,_ which increased by 16.5, 15.5, and 7.3% in comparison with control, respectively. Overall, the augmentation CB, NG, SB, and FC with 10%v/v KKU-MC1 consortium-rich *Bacteroides,* and *Proteobacteria* significantly boosted methane production in terms of potential, rate, yield, and *k*_*h*_ in comparison with non-augmentation. As a result, the MY from CB, NG, SB, and FC augmented with KKU-MC1 was higher than control by 15–42%, 19–29%, 20–32%, and 7–17%, respectively. However, differences in the interaction between the microflora in the original of each substrate and that augmented with KKU-MC1 resulted in different methane production potentials. Many previous studies have applied MC augmentation to improve MY from complex substrates, such as Tukanghan et al. reported that the augmentation with *Bacteroides* and *Clostridium*-rich methanogenic consortium increased MY from palm oil mill effluent and EFB by 86%^13^. Ozbayram et al. found that adding methanogenic consortium-rich *Ruminococcaceae* and *Lachnospiraceae* to the AD reactor increased MY by 27% from wheat straw^61^. Weiss et al. enhanced biogas production from agricultural biomass by 53%, adding *Bacteroides,* *Dechlorosoma*, and *Clostridium*-rich hemicellulolytic consortium^64^. Suksong et al. found that the MY from EFB augmented by *Clostridiaceae*-rich consortium augmentation was 2–10 times higher than non-bioaugmentation^21^. In comparison, the pure culture of *Methanoculleus* *bourgensis* was added to the mesophilic reactor, which caused a stable process and improved microbial metabolism, which increased methane production by 11% ^60^. There have been reported increases in biomethane production of between 8 and 53% when *Caldicellulosiruptor* *lactoaceticus*, *Clostridium* *cellulolyticum*, and hemicellulolytic bacteria have been utilized to target cellulose, wheat straw cellobiose, and xylan, respectively^65^. Higher MY in-augmentation treatment by KKU-MC1 than in the control treatment may be associated with increased synergistic activity between exogenous microorganisms and native AD microorganisms. It has been reported that Bioaugmentation with cellulolytic MC containing synergistic microbiota produces combinations of enzymes for the destruction of lignocellulose and is more resilient to environmental changes and, subsequently, methane production^61,66^. In this study, Bioaugmentation by *Bacteroides* and the *Proteobacteria* rich-KKU-MC1 is the method to increase the synergistic microbial consortia that exhibit powerful lignocellulose degradation capabilities is associated with the rate-limiting phase in the AD process and resulting in increased MY from various biomass. An in-depth study should be performed to identify the interaction and active microorganisms in Bioaugmentation and to compare with non-augmentation. However, due to the difficulty in recovering and reusing the microbial cells and the lack of long-term operating stability, the use of MC for treating biomass in industrial may be limited. The stability of bioprocesses can be improved using hybrid immobilization of microorganisms, which developed based on hybrid entrapment–encapsulation technique, can be further applied in continuous biogas production from lignocellulosic materials.

### Pre-hydrolysis with KKU-MC1

#### Changes in hydrolysate

**Figure 8:**
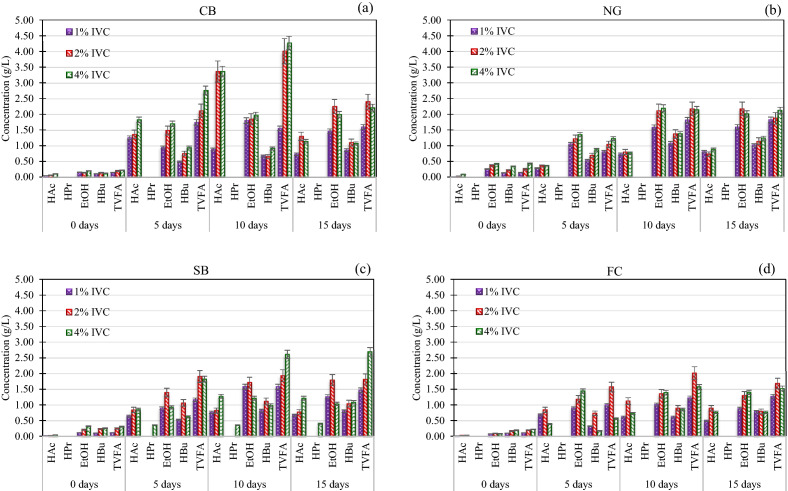
Volatile organic products (VOPs) concentration of cassava bagasse (CB) (**a**), Napier grass (NG) (**b**), sugarcane bagasse (SB) (**c**), and filter cake (FC) (**d**) at 1, 2, and 4% initial volatile solid concentration (IVC) prehydrolyzed by KKU-MC1.

To select the optimal pretreatment time for the CB, NG, SB, and FC with various IVC treated by KKU-MC1, parameters including the sCOD, organic material loss (COD_loss_), rate of COD_loss_ (loss ratio), and VFAs were monitored. The pretreatment of the four lignocellulose materials with KKU-MC1 indicated that a higher IVC resulted in increased VOPs concentrations, as shown in (Fig. 8). The concentration of VOPs in all substrate hydrolysate generally raised progressively throughout the pretreatment process and dropped after the end of the pretreatment. The CB hydrolysate had the maximum VOPs production on day 10, the highest concentration of VOPs was 3.4 ± 0.2, 5.6 ± 0.1, and 6.2 ± 0.3 g/L at 1%, 2%, and 4% of IVC, respectively with predominantly contained HAc, and ethanol (EtOH), followed by a slight decrease on day 15 (Fig. 8a). Similarly, the pH value on day 5 was 5.8, 4.9, and 4.3 at 1%, 2%, and 4% of IVC, respectively, and tended to increase when increased the pretreatment time. VOPs concentrations of NG hydrolysate at 1% IVC tended to increase with increasing pretreatment time and the predominance of EtOH and HBu during days 10 to 15. Similarly, at 2% and 4% IVC of NG hydrolysate, VOPs concentrations increased in a similar range of 4.0–4.3 g/L, where EtOH (2.0–2.2 g/L), and HBu (1.1 –1.4 g/L) were dominant (10–15 days). The NG hydrolysate had a low pH during the initial pretreatment, especially at an IVC of 4%, which corresponded to the VOPs concentrations. Then the pH tended to increase with increasing pretreatment time (Fig. 8b). VOPs concentrations of the SB hydrolysate at 1 and 2% IVC tended to increase initially after day 5, decreased after day 10, and primarily contained EtOH (0.9–1.8 g/L) and HBu (0.5–1.1 g/L). Additionally, at 4% IVC of the SB hydrolysate, 0.4 ± 0.1 g/L of HPr was recorded through the biodegradation of KKU-MC1, while another IVC was not presented (Fig. 8c). Overall, the pH of the SB hydrolysate was comparatively higher than that of the CB, and NG hydrolysates, corresponding to a low VOPs concentration in the hydrolysate. The FC hydrolysate had the lowest VOPs concentration compared to other substrates, corresponding to a relatively high pH value of 6.9–7.2 through the biodegradation of KKU-MC1. VOPs concentrations of all IVC of the FC hydrolysates were increased initially and decreased with increasing pretreatment time. EtOH (1.0–1.4 g/L) and HBu (0.6–0.9 g/L) were the main VOPs (Fig. 8d). The rise in the concentrations of VFAs during the pre-hydrolysis of CB, NG, SB, and FC was undoubtedly caused by the microbial compositions in the KKU-MC1, resulting in a prevalence of HBu, and HAc. The majority of members of KKU-MC1 are Phyla *Bacteroidetes* (genus *Bacteroides* and *Macellibacteroides*), *Proteobacteria* (genus *Citrobacter*), and *Firmicutes* (genus *Clostridium*) (Fig. 3b). These bacteria are responsible for lignocellulose degradation^7,8,36^. The previous study reported that the genus *Bacteroides* belonging to the phylum *Bacteroidetes* has xylanolytic genes to hydrolyze cellulose and hemicellulose in the grass for VFA production ^67^. Moreover, *Macellibacteroides* belonging to the phylum *Bacteroidetes* can metabolize monosaccharides and disaccharides to produce HAc, HBu, and iso-butyric acid (iso-HBu)^40^. The genus *Citrobacter* belonging to the phylum *Proteobacteria* is one of the main microorganisms responsible for the hydrolysis of corn straw to produce VFAs ^68^. Furthermore, the genus *Clostridium* belonging to the phylum *Firmicutes* was the dominant genus in anaerobic fermentation and played a significant role in converting organic substrate to VFAs^69^. Moreover, KKU-MC1 consisted of a class of *Methanobacteria* utilizing these substrates for methane production; hence, KKU-MC1 could synergistically act between cellulolytic and hydrogenotrophic bacteria, which can significantly increase the MY.

**Figure 9:**
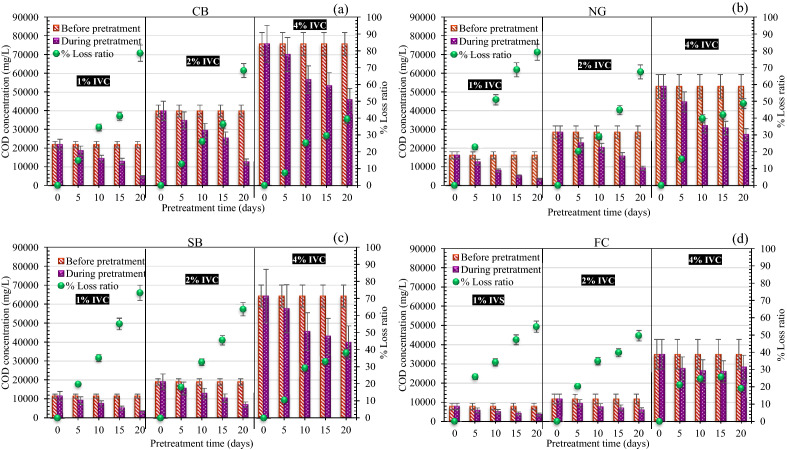
Chemical oxygen demand (COD) balance of cassava bagasse (CB) (**a**), Napier grass (NG) (**b**), sugarcane bagasse (SB) (**c**), and filter cake (FC) (**d**) at 1, 2, and 4% initial volatile solid concentration (IVC) prehydrolyzed by KKU-MC1.

According to Yuan et al., the appropriate time for pretreating the lignocellulose material with varying IVC and pretreatment time must be selected by considering the COD_loss_, and loss ratio^70^. The initial COD concentration of the peptone cellulose solution (PCS) medium (COD_medium_) was 3,855 ± 105.5 mg/L (Fig. 9). In all substrates, an increase in IVC led to an increase in the sCOD_hydrolysate_ concentration at the beginning of the pretreatment period. It then gradually decreased with an increase in pretreatment time. In addition, the CB, NG, and SB had the same tendency for the COD_loss_. The COD_loss_ value was increased with an increase in the pretreatment time. This trend was similar to Yasunori et al.^71^. They reported that more extended biological pretreatment periods resulted in the loss of more organic material during gas production by microbes. Except for FC pretreatment, high sCOD_hydrolysate_ releases and low COD_loss_ with increasing pretreatment time were observed. The CB pretreatment at 4% IVC for 5 days had a high sCOD of 23,500 ± 95.0 mg/L and a low loss ratio of 8%, which was the optimal period for CB pretreatment (Fig. 9a). Similarly, the NG and SB pretreatment at 4% IVC for 5 days showed sCOD concentrations as high as 15,000 ± 125.1, and 12,250 ± 120.0 mg/L, respectively, and a low loss ratio of 16 and 10%, respectively (Fig. 9b,c). Whereas the FC had the lowest concentration of sCOD compared with other substrates, the resulting sCOD corresponds to the concentration of VOPs (Fig. 9d). The FC pretreatment at 4% IVC for 15 days, the sCOD concentration reached 7090 ± 95.5 mg/L and had loss ratio of 26 ± 5.8%. Therefore, we propose that the optimal pretreatment time should be the time with the maximum concentration of sCOD, low COD_loss_, and low loss ratio. The best pretreatment period of CB, NG, and SB with KKU-MC1 was 5 days which was justified by the maximum concentrations of VFAs (Fig. 8) and sCOD (Fig. 9). Our findings coincided with Yuan et al. ^70^ and Ali et al.^34^, who reported that concentrations of sCOD and VFAs rapidly increased to the fourth day of biodegradation of corn stalk and creosote-treated wood biodegradation by microbial consortia, respectively. In contrast, fifteen days of pretreatment were necessary for the FC to release high sCOD. The pretreatment time for CB, NG, and SB with KKU-MC1 was the same duration because the KKU-MC1 were selected from different habitats with plenty of lignocellulosic degrading enzyme and enriched with the lignocellulosic biomass. Meanwhile, the hydrolysis FC with KKU-MC1 required more time (15 days) to digest the insoluble and soluble organic content. In addition, FC contains some difficult-to-degrade substances, such as 9–14% wax and oil and 10–18% resin^72^, which can slow down the degradation rate during hydrolysis, affecting the methane production rate. It was concluded that the optimum pretreatment time for the CB, NG, and SB (at 4% IVC) by KKU-MC1 was 5 days, which showed the highest sCOD, and loss ratio. However, the FC (at 4% IVC) required 15 days of pretreatment to release high sCOD, and low loss ratio.

### Changes in lignocellulosic content and enzyme activity

**Figure 10:**
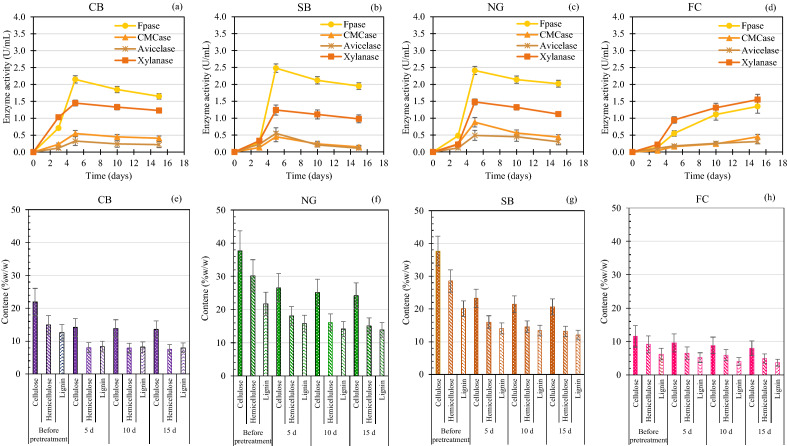
Changes in enzyme activity (**a–d**), and lignocellulose content (**e,f**) of cassava bagasse (CB), Napier grass (NG), sugarcane bagasse (SB), and filter cake (FC) at 1, 2, and 4% initial volatile solid concentration (IVC) prehydrolyzed by KKU-MC1.

At 4% IVC, all substrates were selected for a more detailed study of the weight loss components in the form of cellulose, hemicellulose, and lignin and the activity of the lignocellulosic enzyme. An initial lignocellulosic content of raw CB, NG, SB, and FC were presented in Fig. 10 (at day 0). From the result, it was observed that KKU-MC1 had the highest efficiency in degrading cellulose, hemicellulose, and lignin of the CB, at 35.2 ± 8.5, 46.4 ± 7.5, and 33.8 ± 7.0%, respectively, within 5 days (Fig. 10a). The degradation efficiencies of cellulose, hemicellulose, and lignin of the NG and SB were similar, at 35–41%, 51–57%, and 32–35%, respectively, within 5 days (Fig. 10a,b). Like Wen et al.^12^, who pretreated the NG with MC1, WSD-5, and XDC-2 for 21 days, the loss of cellulose, hemicellulose, and lignin was in the range of 18–22, 30–40, and 30–40%, respectively. In addition, Kong et al. reported that cellulolytic bacteria enriched from composting had the efficiency of degrading cellulose, hemicellulose, and lignin in wheat straw by 30, 65, and 48%, respectively, within 9 days^10^. Weight loss of the three main components revealed that hemicellulose and lignin were mainly broken down by KKU-MC1 within the first 5 days, while cellulose degradation occurred during the entire process. Consistent with the activity of FPase, CMCase, and xylanase increased rapidly after cultivation for 3 days, reaching the maximum values as shown in Fig. 10a–d. FPase, CMCase, and xylanase activity in CB NG and SB hydrolysate presented in the range 2.2–2.5, 0.5–0.9, and 1.2–1.5 IU/mL, respectively. These results further indicated cellulose and hemicellulose degradation in CB, NG, and SB by KKU-MC1. The FC showed low-efficiency degradation of cellulose, hemicellulose, and lignin over 5–10 days. It tended to increase on day 15, when cellulose, hemicellulose, and lignin degradation increased by up to 39.7 ± 8.0, 57.3 ± 9.0, and 35.1 ± 8.2%, respectively (Fig. 10d). Consistent with the activity of FPase, CMCase, and xylanase increased rapidly after cultivation for 10 days that were showed 1.4, 0.5, and 1.6 IU/mL, respectively. While the avicelase activity on all substrates is low. The fastest degradation rates of cellulose, hemicellulose, and lignin in the CB, NG, and SB occurred on day 5, and on days 15, 10, and 15 of the FC, respectively (Fig. 10e–h). From the results, hemicellulose is readily degradable compared to other constituents. According to Wan and Li, MC's first degraded hemicellulose to support microbial growth and metabolism by leaving a cellulose-rich residue^73^. The multi-species lignocellulolytic enzyme in the culture supernatant indicated that KKU-MC1 could significantly improve biogas production from various lignocellulosic materials. It can be concluded that pre-hydrolysis with KKU-MC1 could be released soluble substances (VFAs and sCOD) during the AD process. The corresponding maximum biodegradation efficiency in terms of cellulose and hemicelluloses with KKU-MC1 were 35–46% for the CB, 35–57% for the NG, 41–51% for the SB, and 40–57% for the FC (Fig. 10e–h), respectively, at 4% IVC. It has been previously reported that the cellulose and hemicellulose components of the complex substrate contribute to MY^21^.

### Methane production from hydrolysate

As a result, 4% IVC of all substrates was appropriate for treatment with 10% v/v KKU-MC1. Based on the results, a 5-day pretreatment period was appropriate for the CB, NG, and SB, while the 15-day pretreatment period was appropriate for the FC. Therefore, as mentioned above, the hydrolysate was used as a substrate to study the methane production potential. It was found that the CB that had been pre-hydrolyzed by KKU-MC1 for 5 days had a maximum methane potential of 6,924 ± 95.0 mL-CH4/L (Table 7), 45% greater than the control. In comparison, the NG and SB that were pre-hydrolyzed by KKU-MC1 for 5 days had similar methane production in the range of 5,345–5,677 mL-CH_4_/L, which was increased by 7–14% compared to the NG and SB that had not been pre-hydrolyzed. The methane production of the FC pre-hydrolyzed with KKU-MC1 for 15 days was 2,508.4 ± 105.5 mL-CH_4_/L, which was 20% higher than the control.

**Table 7:** Methane production of lignocellulosic material prehydrolyzed by cellulolytic consortium KKU-MC1.

Treatment	MY (mL-CH_4_/g-VS_added_)	MP (mL-CH_4_/L)	Calculated values from Gompertz model			First order model	
			MPR (mL-CH/g-VS·day)	*k*_*h*_ (day^−1^)	R^2^	Lag phase (day^−1^)	R^2^
5D-4CB	294.6 ± 62.5	6923.6 ± 95.0	12.3 ± 1.0	0.198 ± 0.004	0.990	1.5 ± 0.4	0.981
5D-4NG	286.2 ± 51.2	5676.8 ± 110.5	11.9 ± 3.5	0.112 ± 0.005	0.975	3.5 ± 0.2	0.985
5D-4SB	291.5 ± 23.6	5344.9 ± 98.4	12.2 ± 2.2	0.115 ± 0.005	0.978	4.1 ± 0.1	0.980
15D-4FC	284.0 ± 60.5	2508.4 ± 105.5	11.8 ± 2.5	0.091 ± 0.012	0.995	5.4 ± 0.2	0.994

*5D* substrate pretreated with KKU-MC1 for 5 days, *15D* substrate pretreated with KKU-MC1 for 15 days, *4* at 4% initial volatile solid concentration (IVC).

The MPR of the CB, NG, SB, and FC hydrolysates exhibited a similar range of 11.8–12.3 mL-CH_4_/g-VS·d, which is approximately two times higher than the control of each substrate (Table 7). This finding indicates that the pre-hydrolysis substrate with KKU-MC1 breaks the glycosidic bonds in lignocellulose and increases the hydrolysis efficiency. Therefore, microbes attack the inside of the substrate better than the control treatment (non-prehydrolysis), resulting in an improved MPR. In addition, the pre-hydrolysis strategy accelerated the hydrolysis process in AD, resulting in a shorter lag phase in methane production from CB, NG, SB, and FC pre-hydrolyzed with KKU-MC1 was significantly (p < 0.05) shorter than those of non-hydrolyzed of NG, SB, and FC at all IVC (Table 7). The hydrolysis constant, *k*_*h*_ values of the CB, NG, SB, and FC were 0.198 ± 0.004, 0.112 ± 0.005, 0.115 ± 0.005, and 0.091 ± 0.012 day^−1^, respectively (Table 7), which improved by 1.2–9.6-fold, and 1.2–1.4-fold compared to non-hydrolyzed, and augmented samples, respectively. It was believed that each raw substrate's cellulosic components are challenging to degrade for their smooth surfaces and compact structures. After prehydrolysis with KKU-MC1, these dense structures are degraded, enhancing their interface with hydrolytic bacteria. As a result, the structures are simple for hydrolytic enzymes to break down, increasing the hydrolysis efficiency and k-value^56,74^. The improvement in* k*_*h*_ led to improve MY. MY was 294.6 ± 62.5 mL-CH_4_/g-VS_added_, 53 ± 4.5% higher than the untreated CB (138.9 ± 43.0 mL-CH_4_/g-VS_added_), as shown in Table 7. Moreover, CB pre-hydrolyzed with KKU-MC1 for 5 days prior to the AD process showed MY that was higher by 18.4% compared to the bioaugmentation of KKU-MC1 in the AD process (at 4% IVC). The MY of hydrolyzed NG and SB were 286.2 ± 51.2 and 291.5 ± 23.6 mL-CH_4_/g-VS_added_, respectively, which were in the range 38–43% higher than those of the untreated samples, respectively, and increased by 13–16% in comparison with the augmentation of the KKU-MC1 to the AD system (at 4% IVC). The MY of FC hydrolyzed with KKU-MC1 for 15 days was 284.0 ± 60.5 mL-CH_4_/g-VS_added_, 56% higher than untreated FC (Table 7), and 52% higher than the FC subject to the augmentation method (at 4% VS). The pretreatment of the FC from sugar factories using MCs has not yet been studied. Untreated FC yielded methane in the range of 185.0–208.0 mL-CH_4_/g-VS_added_^75,76^. The findings of this study are consistent with those of other studies in which *Bacteroides, Proteobacteria*, and *Firmicutes* are essential for cellulose degradation^77,78^, and carbohydrate hydrolysis plays a vital role in VFAs degradation^62^. Many previous studies utilized MC rather than enriching various environmental sources active in lignocellulose degradation to enhance biogas production. For example, the maximum MY of pretreated NG by the consortia MC1, WSD-5, and XDC-2 that enriched from the compost materials, plant litter, and composted agricultural and animal wastes, respectively, were 259, 279, 247 mL-CH_4_/g-VS, respectively. These values were 1.39, 1.49, and 1.32 times higher than the untreated material^12^. Thongbunrod et al. reported that the pretreatment of rice straw with the anaerobic lignocellulolytic microbial consortium for 14 days increased MY by 53% compared to untreated rice straw^23^. However, Wen et al. and Thongbunrod et al. used a pretreatment process that required time reached 13–14 days, while this experiment was performed within 5 days. Wongwilaiwalin et al. reported that the pretreatment of NG with promoter library-based module combination at 55 °C for 7 days increased MY by 37% compared to untreated NG^11^. Zhang et al. reported the pretreatment of cassava pulp with MC at 55 °C for 12 h. the MY increased to 259.5 mL-CH_4_/g-VS_added_, more than 97% of the untreated sample^79^. However, according to the study by Wongwilaiwalin and Zhang, using high temperatures for pretreatment may result in higher costs in commercial applications.

Our result can conclude that at 4% IVC of the substrate, the pre-hydrolysis strategy with KKU-MC1 before the AD process was MY higher than the augmentation strategy. This is because KKU-MC1 can destroy the lignin structure, allowing cellulose and hemicellulose to be used by microbes. Additionally, the VFAs, and soluble compounds were released in large quantities in the pre-hydrolysis step. Methanogens rapidly consume these nutrients; hence, methane production in the AD system after pre-hydrolysis and bacterial activity is higher than without pre-hydrolysis^56^. At 1% IVC, the augmentation method obtained an MY that was relatively similar to the pretreatment with KKU-MC1 at 4% IVC. The MY of direct addition of KKU-MC1 to the AD of the CB, SB, and FC were less than those from the pre-hydrolysis method by 4.9, 7.7 and 22.4%. In contrast, upon the direct addition of KKU-MC1 to the AD of the NG at 1% IVC, MY was 7.9% higher than in the pre-hydrolysis method. This indicates that KKU-MC1 augmented by 10%v/v could increase MY at low IVC of the NG and FC better than at high IVC. This may be because the ratio between the MC and the initial organic concentration of the substrate is not appropriate. Therefore, higher loading of KKU-MC1 into the digester is recommended if the IVC of the substrate is above 1%.

## Materials and methods

### Lignocellulosic biomass and inoculum

The SB and FC were obtained from a local sugar industry (Mitr-Phu-Wieng Industry, Khon Kaen, Thailand). The CB was obtained from a local starch industry in Kalasin, Thailand. The Pak Chong 1 strain of the NG was collected from the Department of Animal Science, Faculty of Agriculture, Khon Kaen University, Thailand. It was harvested after 60 days of growth by cutting it 5.0–10.0 cm above the ground. All parts of the NG plant, including stems and leaves, were used as substrates. All dry agricultural waste was crushed in a food processor and sifted through a sieve to a size of < 3 mm. All samples were then sealed in plastic bags and stored at −20 °C until use. Table 1 displays the compositions of all materials. Proximate analyses of biomass substrates were carried out as previously described^80^. The inoculum was obtained from the Up-flow Anaerobic Sludge Blanket (UASB) wastewater treatment plant of a local brewery company in Khon Kaen, Thailand. The inoculum was used with a pH of 7.65 ± 1.2, total solids (TS) of 25.52 ± 7.5%, and VS of 48.13 ± 5.5% for methane production in the AD process. Whatman No. 1 filter paper was used in this study (Whatman, Kent, UK). Chemicals and reagents were of analytical or molecular biology grade and were purchased from Sigma–Aldrich, Fluka, and Merck. All authors confirmed that plant materials were collected according to relevant guidelines, regulations, and legislation.

### Construction of an MC with high lignocellulose degradation capacity

The MC was enriched from distributed areas, including RSC, CM, GD, SGS, RF, and TI. These inoculum sources were collected from the Department of Animal Science, Faculty of Agriculture, Khon Kaen University, Thailand. The TI preparations were prepared according to the method described by Samir Ali et al.^81^. In preliminary screening, one gram of fresh sample was immediately inoculated into a 120 mL serum bottle with 60 mL of PCS medium (0.1% yeast extract, 0.5% peptone, 0.2% CaCO_3_, 0.5% NaCl) containing 2 g of the SB as a complex carbon source during the enrichment procedure. In addition, an extra piece of filter paper (1 × 5 cm) was added as an indicator of cellulase activity. The pH of the medium was adjusted to 7.5 using 5 M NaOH or 5 M HCl solution. Preliminary screening was performed at 37 ºC for 5–8 days. When the strip of filter paper was completely degraded and SB had softened, 10% of each culture was transferred into a fresh PCS medium as mentioned above. This process was repeated 10 times to obtain a stable microbial community capable of degrading the filter paper with high efficiency^82^. The batch subculturing was done in triplicate, and the average results were reported. Afterward, the microorganism sources showing a high filter paper degradation rate were measured and selected^24^.

The filter paper degradation rate was calculated according to the following equations (Eq. 1):

1 $$\mathrm{r}= \frac{{\mathrm{L}}_{0}-{\mathrm{L}}_{\mathrm{p}}}{{\mathrm{T}}_{\mathrm{p}}}$$

where L_o_ (cm) is the original length of the filter paper, L_p_ (cm) is the length of filter paper after pretreatment, and T_p_ (d) stands for the time of pretreatment (time was measured until the filter paper was completely degraded). The removal efficiency of the raw and treated substrate by MC was calculated as follows:

2 $$\mathrm{\% removal \, efficiency }= \frac{{\mathrm{VS}}_{0}}{{\mathrm{VS}}_{\mathrm{t}}}\times {100}$$

where VS is the volatile solid content of the substrate, VS_o_ is the initial concentration of VS at the starting point t = 0, and VS_t_ is the concentration of VS at any time t.

The mixture design with D-optimal from Design-Expert^®^ was applied for the MC construction. In order to design different ratios of three microorganism sources (TI, RSC, and SGS) that gave the highest filter paper degradation rate in fresh PCS medium using SB as a carbon source. Each different ratio was subcultured ten times in PCS medium with SB as a carbon source to obtain a stable microbial community^82^. The batch subculturing was done in triplicate, and the average results were reported.

The MC was named KKU-MC1. Five-day-old MCs were used as inoculum for bio-augmentation in the AD process and to pre-hydrolyze the substrate before the AD process. The MC was maintained in a PCS medium with 20% glycerol (no cellulosic substrate) at −20 °C for long-term storage. The microbial community and the number of genes related to lignocellulose degradation in KKU-MC1 were determined using metagenomics.

### Bioaugmentation using KKU-MC1

Methane production from each lignocellulosic material (CB, SB, NG, and FC) with MC augmentation was investigated in 120 mL serum vials with a working volume of 60 mL. Each vial contained 3% volatile suspended solid (VSS) of methanogenic inoculum and 1%, 2%, or 4% IVC of the substrate, with respective inoculum-to-substrate ratios of 3:1, 3:2, and 3:4. Then, the pH was adjusted to 7.2, using 5 M NaOH or 5 M HCl. Finally, the bottles were adjusted to the working volume with distilled water (dH_2_O). Ten percent (v/v) of KKU-MC1 was added to a serum bottle. There were two controls: the treatments using the 4 substrates without adding KKU-MC1 and the negative control using distilled water as the substrate with the methanogenic inoculum. The headspace of the bottles was purged for 5 min with nitrogen gas to remove oxygen in the head space. Afterward, the bottle was closed with rubber stoppers and an aluminum cap. The serum bottle was incubated in a shaking incubator at 37 °C and 150 rpm. Each vial was treated in quadruplicate, and the average results were reported. Biogas volume was monitored daily using 100 mL glass syringes, and biogas composition was determined using gas chromatography equipped with a thermal conductivity detector (GC-TCD).

### Prehydrolysis of lignocellulosic material with KKU-MC1

The efficiency of KKU-MC1 in decomposing lignocellulosic biomass was evaluated in a 120 mL serum vial. Each vial was prepared by inoculating 6 mL (10% inoculum) of 5-days-old KKU-MC1 into 60 mL of freshly prepared PCS medium containing 0.6, 1.2, and 2.4 g-VS (1, 2, and 4% VS, respectively) of lignocellulosic biomass (CB, SB, NG, and FC). In addition, control experiments with no added KKU-MC1 were included in determining the background activity of microflora in each substrate degradation sample. Each condition was tested in quadruplicate. All vials were subsequently incubated statically at 37 °C.

To investigate the impact of MC pretreatment time on subsequent AD, the experiments were carried out at four different time intervals: 0 (immediately following inoculation), 5, 10, and 15 days. The pretreatment experiment setup consisted of 14 bottles of each substrate. Subsequently, pH, sCOD, COD, VFAs, and weight reduction in cellulose, hemicellulose, and lignin were measured. Optimal IVC (%VS) and pretreatment time were selected based on the obtained high sCOD concentration, and low rate ratio, according to Yuan et al.^70^.

### The hydrolysate of lignocellulosic material pretreated with KKU-MC1 for biogas production

The biomethane potential (BMP) test consists of the methanogenic inoculum, and the substrates CB, SB, NG, and FC hydrolysates (including residual each substrate and fermentation broth) at a 3:1 ratio based on VSS: VS was investigated in 120 mL serum vials with a working volume of 60 mL. For comparison, in terms of MY and degradation efficiency, raw CB, SB, NG, and FC with methanogenic inoculum were used as the control. The pH was adjusted to 7.2 with either 5 M HCl or 5 M NaOH. The bottle's headspace was purged with nitrogen gas to remove oxygen in the headspace for 5 min. Afterward, the bottle was closed with rubber stoppers, followed by an aluminum cap, placed in an incubator at 37 °C, and shaken at 150 rpm. An inoculum containing water was used as a negative control instead of the samples. Each vial was treated in quadruplicate, and the average results were reported. Biogas volume was monitored daily using 100 mL glass syringes, and biogas composition was determined using GC-TCD.

### Metagenomics analysis

Genomic DNA (gDNA) was extracted from the samples at 300–500 ng/mL concentrations and submitted for metagenomic sequencing to Novogene Co., Ltd. (Beijing, China). Whole metagenomic shotgun sequencing was performed using the Illumina Hi-Seq2500 V4 PE125 (paired-end 150 bp) platform. Briefly, the whole gDNA was treated using a next-generation sequencing Fast DNA Library Prep Set for Illumina Hi-Seq 2500 V4 PE125 to prepare the metagenomic library following a standard Illumina protocol. The raw sequences were initially processed by removing low-quality reads (reads containing N nucleotides > 10%, reads containing adaptor sequences, and reads where 40% of the bases had a Q score of 38). Clean reads were assembled using SOAPdenovo^83^, and reads with the default parameters (k-mer size of 55 and scaftigs less than 500 bp) were kept for further analysis. Genes were predicted using MetaGeneMark from the scaftigs. After dereplication, all unique genes were used to construct the gene catalogue. BLAST was performed against the MicroNR database to generate taxonomy annotation information for the gene catalog. The top 10 taxa, including phylum, class, order, family, and genus, were visually shown using Krona^84^. The functional annotation databases were used: the KEGG^85–87^, eggNOG (Version: 4.1)^88^, and CAZymes (Version: 2014.11.25)^89^. To represent the number of genes coding for lignocellulose degrading enzymes with the most abundant genus found in the KKU-MC1 metagenome. We have used Microsoft Excel 365 to visualize heat maps and Microsoft PowerPoint 365 to visualize pathways.

### Analytical methods

The TS, VS, and ash values of the lignocellulosic materials (CB, SB, NG, and FC), and methanogenic inoculum were analyzed according to the Standard Methods for the Examination of Water and Wastewater^80^. The pH, VOPs, COD of substrate (COD_substrate_) before pre-hydrolysis, and the COD of residue (COD_residue_) during pre-hydrolysis were analyzed using extracted solution. To obtain the extracted solution, 5 g of each lignocellulose material was homogenized in 45 mL of dH_2_O and stirred at 150 rpm for 4 h. Then extracted solution was centrifuged at 15,000*g* for 5 min and filtered through a 0.45 mm filter before analysis. The supernatants from hydrolysate, and PCS medium were measured with a Spectroquant^®^ COD cell test kit (Merck, Germany) for sCOD, and COD_medium_, respectively. The COD balance analysis during pre-hydrolysis was calculated^70^. The COD_loss_ was calculated using Eqs. (3) and (4):

3 $${\mathrm{COD}}_{\mathrm{loss}}\hspace{0.17em}=\hspace{0.17em}[{\mathrm{COD}}_{\mathrm{substrate}}{+\mathrm{COD}}_{\mathrm{medium}}]-\left[{\mathrm{COD}}_{\mathrm{residue}}\hspace{0.17em}+\hspace{0.17em}{\mathrm{sCOD}}_{\mathrm{hydrolysate}}\right]$$

The loss ratio of organic materials was calculated as follows:

4 $$\mathrm{Loss\, Ratio}=\left[\frac{{\mathrm{COD}}_{\mathrm{loss}}}{\left({\mathrm{COD}}_{\mathrm{substrate}}{+\mathrm{COD}}_{\mathrm{medium}}\right)}\times 100\mathrm{\%}\right]$$

The pH was measured using a pH meter (pH-500, Queen, USA). The concentrations of VOPs, that is, HAc, HPr, HBu, and EtOH, were determined using high-performance liquid chromatography (HPLC) (Shimadzu LC-20AD, Japan) with a refractive index detector (RID). A 7.80 × 300 mm Vertisep™ OA column (Vertical Chromatography, Thailand) was used. The oven temperature was set at 45 °C, and 5 mM sulfuric acid at a flow rate of 0.5 mL/min was used as the mobile phase^90^. The total VFAs were the sum of HAc, HPr, and HBu during prehydrolysis. Monosaccharide components were quantified by HPLC (Shimadzu LC-20AD, Japan) using an Aminex HPX-87H column (Bio-Rad, USA) equipped with a RID-10A (Shimadzu, Japan). HPLC conditions were detailed by Nualsri et al.^91^. The volume of biogas was measured using a wetted glass syringe of 5–50 mL. Gas samples were analyzed for methane content using a GC-TCD. A 2-m stainless column packed with Shin carbon (50/80 mesh) was used. Helium gas was used as carrier gas. The conditions of the GC-TCD were set according to Pattra et al.^92^. The total volume of methane was produced using the mass balance equation The volume of methane was calculated using the mass balance equation^93^.

The cellulose, hemicellulose, and lignin contents of the untreated and pretreated materials were determined following the National Renewable Energy Laboratory (NREL) protocol^94^. The enzyme activity in the hydrolysate was evaluated in this study. Briefly, the hydrolysates of different lignocellulosic materials were centrifuged at 12,000*g* for 10 min, and the supernatant was collected and used for enzyme assays. One milliliter of properly diluted supernatant was mixed with 1 mL pre-warmed citrate buffer (pH 6.0, 0.1 M), containing a 1.0 × 6.0 cm. Whatman no. 1 filter paper, 1% (w/v) carboxymethyl cellulose, 1% (w/v) microcrystalline cellulose Avicel^®^, and 1% (w/v) of beech wood xylan as the substrates for determining the enzyme activity of FPase, CMCase, avicelase, and xylanase, respectively. The reaction was incubated at 60 °C for 60 min. The amount of reducing sugars was determined by the dinitrosalicylic (DNS) acid method using absorbance measurements at 540 nm and interpolated from a standard curve of the corresponding sugars^95^. One unit of FPase, CMCase, or avicelase activity was defined as the amount of enzyme that produced 1 µmol of reducing sugar per minute. In comparison, one unit of xylanase activity was defined as the amount of enzyme that released 1 µmol of xylose per minute under assay conditions.

The mixture design with D-optimal from Design-Expert^®^ trial version 7.0 software (Stat-Ease Inc., Minneapolis, MN, USA) was applied to determine the optimum proportions of RSC (X_1_), SGS (X_2_) and TI (X_3_) that maximized the enzyme activity (FPase, avicelase, CMCase, and xylanase) and maximized the VS removal efficiency. Mixtures were designed with varying proportions of cellulolytic microbial sources at a total volume of 3 mL (Table 3). Eleven experimental points of each component were expressed in mL and the sum of all components (X_1_ + X_2_ + X_3_) was added up to 3 mL. Different models, including linear, quadratic, and cubic, were used to analyze the mixture proportions resulting in the maximum enzyme activity or VS removal efficiency. In previous work, each model's equations utilized in the study were published^76^. A combination of RSC, SGS, and TI was investigated for synergistic or antagonistic effect in each proportion of microorganism source by experimental enzyme activity (including FPase, CMCase, Avicelase, and Xylanase) or VS removal efficiency and calculated enzyme activity or VS removal efficiency. Experimental enzyme activity or VS removal efficiency is a value obtained from the experiment. The calculated enzyme activity or VS removal efficiency is calculated based on the individual substrates (based on mL) contained in the proportion. The synergistic effect and antagonistic effect of the combination were calculated as the experimental and calculated enzyme activity or VS removal efficiency ratio. An antagonistic effect is indicated by a ratio less than 1, whereas a synergistic effect is indicated by a ratio higher than 1. A value of 1 means there is no effect^96,97^.

A modified Gompertz model was used to fit the cumulative methane production curves^98^, which are shown as Eq. (5)

5 $$M(t)=P\times \mathrm{exp}\left\{-\mathrm{exp}\left[\frac{{R}_{max } \times e}{\mathrm{P}} \left[\left(\lambda -\mathrm{t}\right)+1\right]\right]\right\}$$

where *M* is the cumulative methane production (mL/L) at a given time t (days); *P* is the methane production potential (mL/L); *R*_*max*_ is the specific methane production rate (mL-CH_4_/g-VS_added_^**.**^ days); *λ* is the lag phase time (days); *t* is the duration of the assay (days), and *e* equal to 2.71828. The parameters of *P,* *R*_*m*ax_, and *λ* were estimated using a non-linear curve fitting in SigmaPlot 11 (Systat Software Inc., USA). The MYs were calculated by the methane production potential (mL/L) divided by VS_added_ and expressed in unit mL-CH_4_/g-VS_added_. The constant for the hydrolysis rate, *k*_*h*_, was determined considering the first-order model described in Angelidaki et al. ^99^. Statistical analyses were performed using SPSS Statistics 25 software (SPSS, Inc., Chicago, IL, USA). One-way ANOVA was performed, followed by Duncan's multiple range tests with statistical significance set at p-value < 0.05. All authors confirmed that the methods were performed following relevant guidelines, regulations, and legislation.

## Conclusions

The KKU-MC1 is dominated by taxa belonging to the phyla *Bacteroidetes,* *Proteobacteria,* and *Firmicutes*, as shown by the metagenomic sequences. Diverse members of *Bacteroidetes,* and *Firmicutes* secreted a variety of cellulose, and hemicellulose degradation-related enzymes, including endoglucanase, -glucosidase, -galactosidase, and -xylosidase. GMC family oxidoreductase, and multicopper oxidase secreted by *Proteobacteria* contribute to lignin degradation. Therefore, KKU-MC1 improves biogas production from lignocellulose materials. Augmentation with KKU-MC1 led to a richer and more diverse microbial community and improved the MY of the CB, NG, SB, and FC in the range of 7–42% at 1, 2, and 4% IVC compared to non-augmentation. Furthermore, the pre-hydrolyzed CB, NG, SB, and FC at 4% IVC with KKU-MC1 led to enhanced soluble substances for the AD process, and FC increased the MY by about 38–56% compared to the raw material. These results demonstrate that KKU-MC1 has the potential to decompose various lignocellulosic biomasses, which results in improved biogas production.

## Supplementary Information

Supplementary Figure S1.

Supplementary Table S1.

Supplementary Table S2.

Supplementary Table S3.

Supplementary Table S4.

Supplementary Table S5.

## Data Availability

The authors confirm that the data supporting the findings of this study are available within the article.
