# Supplementary material for: Taxonomic and enzymatic basis of the cellulolytic microbial consortium KKU-MC1 and its application in enhancing biomethane production
Source: Sci Rep. 2023 Feb 20;13:2968. doi: 10.1038/s41598-023-29895-0 (PMC9941523; doi:10.1038/s41598-023-29895-0)
Supplement: Supplementary file 3 — Supplementary Table S2. [file 41598_2023_29895_MOESM3_ESM.docx]

**Taxonomic and enzymatic basis of the cellulolytic microbial consortium KKU-MC1 and its application in enhancing biomethane production**

Nantharat Wongfaed^1^, Sompong O-Thong^2^, Sureewan Sittijunda^3^ & Alissara Reungsang^1,4,5,*^

^1^Department of Biotechnology, Faculty of Technology, Khon Kaen University, Khon Kaen, 40002, Thailand

^2^International College, Thaksin University, Songkhla, 90000, Thailand

^3^Faculty of Environment and Resource Studies, Mahidol University, Nakhon Pathom, Thailand

^4^Research Group for Development of Microbial Hydrogen Production Process from Biomass, Khon Kaen University, Khon Kaen, 40002, Thailand

^5^Academy of Science, Royal Society of Thailand, Bangkok, Thailand

*Corresponding author : Alissara Reungsang

alissara@kku.ac.th

Department of Biotechnology, Faculty of Technology, Khon Kaen University, Khon Kaen, 40002, Thailand

Research Group for Development of Microbial Hydrogen Production Process from Biomass, Khon Kaen University, Khon Kaen, 40002, Thailand

Academy of Science, Royal Society of Thailand, Bangkok, Thailand

**Table S2** Analysis of variance (ANOVA) for the mixture quadratic and cubic model.

| FPase |  |  |  |  |  |
| --- | --- | --- | --- | --- | --- |
| Source | Sum of Squares | degree of freedom | Mean square | F-Value | p-value Prob > F |
| Model | 5.72 | 5.00 | 1.14 | 4.7 | 0.027 |
| Linear Mixture | 0.23 | 2.00 | 0.12 | 0.5 | 0.640 |
| AB | 0.23 | 1.00 | 0.23 | 0.9 | 0.361 |
| AC | 5.44 | 1.00 | 5.44 | 22.3 | 0.002 |
| BC | 0.01 | 1.00 | 0.01 | 0.0 | 0.864 |
| Residual | 1.95 | 8.00 | 0.24 |  |  |
| Lack of Fit | 1.94 | 5.00 | 0.39 | 202.0 | 0.001 |
| Pure Error | 0.01 | 3.00 | 0.00 |  |  |
| Cor Total | 7.67 | 13.00 |  |  |  |
| R-Squared | 0.750 |  |  |  |  |

| CMCase |  |  |  |  |  |
| --- | --- | --- | --- | --- | --- |
| Source | Sum of Squares | degree of freedom | Mean square | F-Value | p-value Prob > F |
| Model | 0.05 | 5.00 | 0.01 | 4.3 | 0.034 |
| Linear Mixture | 0.03 | 2.00 | 0.02 | 6.0 | 0.026 |
| AB | 0.01 | 1.00 | 0.01 | 4.5 | 0.068 |
| AC | 0.00 | 1.00 | 0.00 | 0.5 | 0.516 |
| BC | 0.01 | 1.00 | 0.01 | 3.9 | 0.084 |
| Residual | 0.02 | 8.00 | 0.00 |  |  |
| Lack of Fit | 0.02 | 5.00 | 0.00 | 117.1 | 0.001 |
| Pure Error | 0.00 | 3.00 | 0.00 |  |  |
| Cor Total | 0.07 | 13.00 |  |  |  |
| R-Squared | 0.730 |  |  |  |  |

| Avicelase |  |  |  |  |  |
| --- | --- | --- | --- | --- | --- |
| Source | Sum of Squares | degree of freedom | Mean square | F-Value | p-value Prob > F |
| Model | 0.05 | 5.00 | 0.01 | 5.7 | 0.016 |
| Linear Mixture | 0.03 | 2.00 | 0.01 | 8.1 | 0.012 |
| AB | 0.01 | 1.00 | 0.01 | 6.0 | 0.040 |
| AC | 0.00 | 1.00 | 0.00 | 0.2 | 0.662 |
| BC | 0.01 | 1.00 | 0.01 | 5.0 | 0.055 |
| Residual | 0.01 | 8.00 | 0.00 |  |  |
| Lack of Fit | 0.01 | 5.00 | 0.00 |  |  |
| Pure Error | 0.00 | 3.00 | 0.00 |  |  |
| Cor Total | 0.06 | 13.00 |  |  |  |
| R-Squared | 0.780 |  |  |  |  |

| Xylanase |  |  |  |  |  |
| --- | --- | --- | --- | --- | --- |
| Source | Sum of Squares | degree of freedom | Mean square | F-Value | p-value Prob > F |
| Model | 0.82 | 6.00 | 0.14 | 5.8 | 0.018 |
| Linear Mixture | 0.45 | 2.00 | 0.22 | 9.5 | 0.010 |
| AB | 0.07 | 1.00 | 0.07 | 3.0 | 0.128 |
| AC | 0.28 | 1.00 | 0.28 | 11.8 | 0.011 |
| BC | 0.01 | 1.00 | 0.01 | 0.4 | 0.527 |
| ABC | 0.13 | 1.00 | 0.13 | 5.5 | 0.052 |
| Residual | 0.17 | 7.00 | 0.02 |  |  |
| Lack of Fit | 0.16 | 4.00 | 0.04 | 21.300 | 0.015 |
| Pure Error | 0.01 | 3.00 | 0.00 |  |  |
| Cor Total | 0.98 | 13.00 |  |  |  |
| R-Squared | 0.832 |  |  |  |  |

| VS removal efficiency |  |  |  |  |  |
| --- | --- | --- | --- | --- | --- |
| Source | Sum of Squares | degree of freedom | Mean square | F-Value | p-value Prob > F |
| Model | 459.69 | 6.00 | 76.61 | 5.74 | 0.0185 |
| Linear Mixture | 274.14 | 2.00 | 137.07 | 10.28 | 0.0083 |
| AB | 10.45 | 1.00 | 10.45 | 0.78 | 0.4054 |
| AC | 46.52 | 1.00 | 46.52 | 3.49 | 0.1041 |
| BC | 9.41 | 1.00 | 9.41 | 0.71 | 0.4287 |
| ABC | 105.43 | 1.00 | 105.43 | 7.90 | 0.0261 |
| Residual | 93.38 | 7.00 | 13.34 |  |  |
| Lack of Fit | 75.38 | 4.00 | 18.84 | 3.14 | 0.1871 |
| Pure Error | 18.00 | 3.00 | 6.00 |  |  |
| Cor Total | 553.06 | 13.00 |  |  |  |
| R-Squared | 0.830 |  |  |  |  |
