# Supplementary material for: Taxonomic and enzymatic basis of the cellulolytic microbial consortium KKU-MC1 and its application in enhancing biomethane production
Source: Sci Rep. 2023 Feb 20;13:2968. doi: 10.1038/s41598-023-29895-0 (PMC9941523; doi:10.1038/s41598-023-29895-0)
Supplement: Supplementary file 5 — Supplementary Table S4. [file 41598_2023_29895_MOESM5_ESM.docx]

**Taxonomic and enzymatic basis of the cellulolytic microbial consortium KKU-MC1 and its application in enhancing biomethane production**

Nantharat Wongfaed^1^, Sompong O-Thong^2^, Sureewan Sittijunda^3^ & Alissara Reungsang^1,4,5,*^

^1^Department of Biotechnology, Faculty of Technology, Khon Kaen University, Khon Kaen, 40002, Thailand

^2^International College, Thaksin University, Songkhla, 90000, Thailand

^3^Faculty of Environment and Resource Studies, Mahidol University, Nakhon Pathom, Thailand

^4^Research Group for Development of Microbial Hydrogen Production Process from Biomass, Khon Kaen University, Khon Kaen, 40002, Thailand

^5^Academy of Science, Royal Society of Thailand, Bangkok, Thailand

*Corresponding author : Alissara Reungsang

alissara@kku.ac.th

Department of Biotechnology, Faculty of Technology, Khon Kaen University, Khon Kaen, 40002, Thailand

Research Group for Development of Microbial Hydrogen Production Process from Biomass, Khon Kaen University, Khon Kaen, 40002, Thailand

Academy of Science, Royal Society of Thailand, Bangkok, Thailand

| **Functional Category** | **Gene Num** |
| --- | --- |
| A: RNA processing and modification | 7 |
| B: Chromatin structure and dynamics | 27 |
| C: Energy production and conversion | 5136 |
| D: Cell cycle control, cell division, chromosome partitioning | 700 |
| E: Amino acid transport and metabolism | 6619 |
| F: Nucleotide transport and metabolism | 1960 |
| G: Carbohydrate transport and metabolism | 6467 |
| H: Coenzyme transport and metabolism | 2351 |
| I: Lipid transport and metabolism | 1663 |
| J: Translation, ribosomal structure and biogenesis | 3952 |
| K: Transcription | 4517 |
| L: Replication, recombination and repair | 4383 |
| M: Cell wall/membrane/envelope biogenesis | 4880 |
| N: Cell motility | 695 |
| O: Posttranslational modification, protein turnover, chaperones | 2410 |
| P: Inorganic ion transport and metabolism | 5164 |
| Q: Secondary metabolites biosynthesis, transport and catabolism | 790 |
| S: Function unknown | 24537 |
| T: Signal transduction mechanisms | 3348 |
| U: Intracellular trafficking, secretion, and vesicular transport | 1171 |
| V: Defense mechanisms | 1582 |
| W: Extracellular structures | 3 |
| Z: Cytoskeleton | 1 |
| Others | 32822 |

**Table S2.** The detailed Clusters of Orthologous Groups of protein (COG) classification of KKU-MC1.
